# Genetic diversity and population structure analyses of tropical maize inbred lines using Single Nucleotide Polymorphism markers

**DOI:** 10.1371/journal.pone.0315463

**Published:** 2025-01-24

**Authors:** Rodreck Gunundu, Hussein Shimelis, Seltene Abady Tesfamariam

**Affiliations:** 1 African Centre for Crop Improvement (ACCI), College of Agriculture, Engineering and Science (CAES), University of KwaZulu-Natal, Scottsville, Pietermaritzburg, South Africa; 2 Seed Co, Rattray Arnold Research Station, Harare, Zimbabwe; Government College University Faisalabad, PAKISTAN

## Abstract

Analyses of the genetic distance and composition of inbred lines are a prerequisite for parental selection and to exploit heterosis in plant breeding programs. The study aimed to assess genetic diversity and population structure of a maize germplasm panel comprising 182 founder lines and 866 derived inbred lines using Single Nucleotide Polymorphism (SNP) markers to identify genetically unique lines for hybrid breeding. The founder lines were genotyped with 1201 SNPs, and the derived lines with 1484 SNPs. Moderate genetic variation, with genetic diversity ranging from 0.004 to 0.44 with a mean of 0.25, was recorded for the founder lines, while corresponding values of 0.004 to 0.34 with a mean of 0.13 were recorded for the derived lines. Heterozygosity values ranging from 0.00 to 0.24 and a mean of 0.08 were recorded for both lines. Of the SNP markers used, 82% of the 1201 markers and 84% of the 1484 markers exhibited polymorphism information content ranging from 0.25 to 0.50. Analysis of molecular variance revealed significant genetic differences (P ≤ 0.001) among and within populations in the founder and derived lines. Most detected variations, i.e., 97% and 88.38%, were attributed to within populations in the founder and derived lines, respectively. Population structure analysis identified three distinct subpopulations among founder lines and two among derived lines. Cluster analysis supported the population structure The following genetically distant founder and derived inbred lines were selected: G15NL337 and G15NL312 (Cluster 1), 15ARG152 and RGS-PL44 (Cluster 2), RGS-PL44 and 15ARG149 (Cluster 2), and RGS-PL33 and RGS-PL44 (Cluster 2), respectively. The selected lines are genetically distinct and recommended for marker-assisted hybrid maize breeding to exploit the frequency of beneficial alleles. This study provides valuable insights for maize breeding programs, enabling the exploitation of beneficial alleles and contributing to improved crop yields and food security through hybrid breeding.

## Introduction

Globally, maize (*Zea mays* L., 2n = 2x = 20) is the second most widely cultivated cereal crop after wheat, with an estimated production area of 204 million hectares and 1,163 million tonnes of grain production annually [1]. Maize plays a pivotal role in food security and global economies. It is the leading staple food in developing countries and a crucial raw material for the livestock and processing industries [2, 3]. Maize is a strategic crop in Africa’s food systems, providing approximately 30% of the continent’s energy intake. In Africa, smallholder farmers produce maize and trade it in local markets for household food security and cash income.

South Africa is the leading maize producer in Africa, with a production of 16.1 million metric tonnes per annum, and ranks as the world’s ninth largest maize exporter after the USA, Brazil, Argentina, Ukraine, Romania, France, Paraguay and Poland [4, 5]. Other important maize producers in Africa are Nigeria (12.9 million tonnes of grain per year), Ethiopia (10.2 million tonnes), and Egypt (7.5 million tonnes) [1]. The total maize production in Africa is 93 million tonnes annually, far below the demand of approximately 150 million tonnes. The grain deficit is met through imports notably from Argentina, Ukraine and Brazil. In 2020, Argentina was the top maize exporter to Africa, accounting for a monetary value of $1.8 billion, followed by Ukraine ($856 million) and Brazil ($824 million) [1].

Africa accounts for only 8% of the global maize production. The mean maize grain yield in the region is low at 2.1 t/ha compared to the worldwide average of 5.8 t/ha. This yield gap is attributed to a combination of abiotic stresses (e.g., heat and drought stress, flooding, waterlogging, poor soil fertility, and soil erosion) [6–9] and biotic stresses, including plant diseases (e.g., grey leaf spot, maize streak virus, maize lethal necrosis disease, *Phaesopharia* leaf spot and northern corn leaf blight), parasitic weeds (e.g. *Striga* species) and insect pests (e.g., the fall armyworm, stem borers, cutworms, termites and leafhoppers). These stresses contribute to substantial yield losses and crop failure in the region [3, 10]. Therefore, genetic innovations and modern production technologies are crucial to improving the yield potential and closing the yield gap.

Hybrid maize breeding is critical in developing resilient, locally adapted, and high-performing cultivars. Hybrids achieve increased yields, withstand diseases and pests more effectively, and provide better nutritional content. Furthermore, they demonstrate exceptional resilience to drought, heat, flooding, and poor soil quality [11]. The success of hybrid breeding depends on heterosis, or hybrid vigour, which is maximized by selecting genetically distant and contrasting inbred lines [12].

Genetic variation allows for the selection of favourable genetic combinations among different and complementary parents [13–16]. A well-characterized genetic resource is essential for identifying potential parents, heterotic groups, and guiding conservation [17–19]. Analyses of genetic structure and diversity provide valuable insights into the relationships between breeding lines, which can guide hybrid breeding [20, 21]. Several studies have assessed the population structure and genetic diversity among varied maize populations and contrasting test environments and marker systems. For instance, Lu et al. [13] analyzed 770 maize lines, identifying distinct population structures and genetic divergence between temperate and subtropical/tropical germplasm using 1,034 SNPs. Yan et al. [22] reported well-delineated genetic structures between temperate and tropical lines among 632 inbred lines. Adu et al. [20] assessed genetic diversity in 94 tropical maize inbred lines, clustering them by pedigree, selection history, and endosperm color. Furthermore, Wen et al. [23] examined 359 maize inbred lines developed by CIMMYT and IITA, displaying variable tolerance to abiotic and biotic stresses. The present and past findings indicate the need for rigorous genetic diversity analysis of candidate test populations using high throughput SNP markers to appraise the genetic structure and lineage and guide selection and breeding. Notably, hybrids developed from diverse heterotic groups consistently outperform their parents in grain yield and yield components traits [24].

Elite inbred lines can be assigned to distinct heterotic groups using phenotyping, pedigree analyses, and genetic distance estimates [20]. Morphological, biochemical, and molecular markers are commonly used in genetic diversity analysis and genetic grouping [25]. DNA markers (e.g., Single Nucleotide Polymorphisms (SNPs), Simple Sequence Repeats (SSRs), Restriction Fragment Length Polymorphisms (RFLPs), Randomly Amplified Polymorphic DNAs (RAPDs), and Amplified Fragment Length Polymorphisms (AFLPs)) have become complementary tools to phenotyping tools. Genetic markers have high repeatability with limited influence from genotype x environment interaction effects. SNPs have become the preferred choice of markers to genotype maize populations due to their low cost per data point, widespread presence in the genome, specific location at genetic loci, co-dominance, amenable for high-throughput analysis, and lower rates of genotyping errors [26, 27]. SNPs have been used to identify distinct subpopulations [28], determine genetic diversity within and between landraces [29], assess genetic diversity of early maturing white and yellow tropical maize inbred lines [20], determine the rate of decay of linkage disequilibrium [30], and discern population structure [31].

Analyses of the genetic distance and composition of inbred lines are a prerequisite for parental selection and to exploit heterosis in hybrid breeding programs. Seed Co Ltd systematically bred and selected founder and derived elite maize inbred lines from two major heterotic groups to develop high-performing commercial single cross and three-way hybrids. However, there is lack of information on the genetic diversity and relationship of these lines to guide the regional maize breeding program. In this regard, the test lines should be characterized with diagnostics SNP markers to select genetically distinct and complimentary lines for marker-assisted hybrid maize breeding to exploit the frequency of beneficial alleles. Therefore, this study aimed to assess the genetic diversity and population structure of 182 founder lines and 866 derived inbred lines of maize using SNP markers to identify genetically complementary lines for hybrid breeding.

## Materials and methods

### Plant material

The study used 182 elite founder-inbred lines and 866 derived inbred lines of maize from tropical and subtropical genetic lineages. The founder lines, sourced from the Seed Co Ltd maize germplasm pool, are elite parental lines selected to develop improved maize varieties. These lines are widely utilized in breeding programs and are prominent in most released hybrid varieties in Zimbabwe. The 866 tropical inbred lines were developed from diverse source populations created by crossing founder lines, selected for their adaptability to tropical and subtropical environments in sub-Saharan Africa (SSA) after rigorous testing. The lines were selected based on desirable agronomic characteristics, such as high yield potential, drought tolerance, and disease resistance. Table 1 summarises the list of lines and their respective heterotic groups [N3 (group 1) and SC (group 2)]. The N3 heterotic group were originated from the Salisbury white landrace, which was cultivated in Salisbury (now known as Harare) before the introduction of hybrid maize in 1960. On the other hand, the SC group was obtained from a landrace grown on Mr Southey’s farm and was named "Southern Cross". The N3 was specifically designated as "Northern Cross" to highlight the contrast with the SC inbreds.

**Table 1:** List of the founder and derived lines used in the study.

Inbred lines	Code or designation	Description
Founder lines	RGS-PL65, RGS-PL08, RGS-PL09, RGS-PL68, RGS-PL63, RGS-PL62, RGS-PL32, RGS-PL38, RGS-PL53, RGS-PL20, RGS-PL10, RGS-PL58, RGS-PL06, 15ARG119, 15ARG142, 15ARG143, 15ARG148, 15ARG149, 15ARG151, 15ARG175, RGS-PL48, RGS-PL52, 15ARG104, 15ARG176, RGS-PL54, RGS-PL19, RGS-PL17, 15ARG112, 15ARG127, 15ARG129, 15ARG152, RGS-PL60, RGS-PL13, RGS-PL07, 15ARG111, 15ARG114, 15ARG132, 15ARG140, 15ARG161, RGS-PL66, RGS-PL11, RGS-PL36, RGS-PL01, RGS-PL44, 15ARG110, 15ARG123, 15ARG164, RGS-PL43, RGS-PL47, RGS-PL69, 15ARG117, 15ARG128, 15ARG158, 15ARG160, 15ARG173, RGS-PL50, RGS-PL03, RGS-PL59, 15ARG131, 15ARG137, 15ARG147, 15ARG154, 15ARG155, 15ARG159, 15ARG165, 15ARG167, 15ARG174, RGS-PL18, RGS-PL24, 15ARG106, 15ARG107, 15ARG116, 15ARG125, 15ARG126, 15ARG130, 15ARG144, 15ARG153, 15ARG171, RGS-PL05, RGS-PL45, 15ARG121, 15ARG124, 15ARG157, 15ARG168, RGS-PL64, RGS-PL56, RGS-PL55, RGS-PL15, RGS-PL21, RGS-PL02, 15ARG145, 15ARG163, 15ARG172, 15ARG109, 15ARG120, 15ARG122, 15ARG136, 15ARG138, 15ARG166, 15ARG177, RGS-PL14, RGS-PL16, RGS-PL40, 15ARG113, 15ARG115 15ARG150 15ARG156 RGS-PL57 RGS-PL61 RGS-PL49 RGS-PL37 RGS-PL12, RGS-PL22, RGS-PL0, RGS-PL39, 15ARG105, 15ARG133, 15ARG135, 15ARG139, 15ARG169, RGS-PL70, RGS-PL42, 15ARG103, 15ARG146, 15ARG170, RGS-PL67, RGS-PL41, 15ARG108, 15ARG118, 15ARG134, 15ARG141, 15ARG162	Heterotic group 1 (N3)
	16ARG16793, 16ARG16799, 16ARG16808, RGS-PL46, RGS-PL28, 16ARG16812, 16ARG16786, 16ARG16788, 16ARG16791, 16ARG16792, 16ARG16804, 16ARG16816, RGS-PL35, RGS-PL30, 16ARG16798, 16ARG16815, 16ARG16782, 16ARG16814, RGS-PL71, 16ARG16784, 16ARG16795, RGS-PL25, RGS-PL31, RGS-PL29, 16ARG16794, 16ARG180, 16ARG16790, 16ARG16802, 16ARG16806, 16ARG16811, RGS-PL33, 16ARG178, 16ARG16803, 16ARG16807, RGS-PL27, 16ARG16781, 16ARG16783, RGS-PL34, 16ARG16789, 16ARG16796, 16ARG16797, 16ARG16801, 16ARG16805, 16ARG181, 16ARG16800, 16ARG179, 16ARG16787, 16ARG16810, 16ARG16813, 16ARG16785, 16ARG16809, 16ARG1681, RGS-PL26	Heterotic group 2 –(SC)
Derived lines	G15NL01—G15NL12; G15NL14—G15NL20; G15NL22—G15NL27; G15NL29—G15NL36; G15NL281; G15NL283—G15NL314; G15NL316—G15NL321; G15NL323—G15NL334; G15NL336—G15NL342; G15NL344—G15NL357; G15NL359—G15NL365; G15NL367—G15NL370, G16NL37—G16NL50; G16NL54—G16NL68; G16NL70—G16NL74; G16NL77—G16NL83; G16NL85—G16NL99; G16NL100—G16NL109; G16NL112—G16NL138; G16NL141—G16NL145; G16NL147—G16NL153; G16NL155, G16NL156, GL17NL157—G17NL170; G17NL172—G17NL180; G17NL182, G17NL226; G17NL228—G17NL239; G17NL241—G17NL242, G18NL206, G18NL240—G18NL246; G18NL248—G18NL254; G18NL257—G18NL267; G18NL269—G18NL271; G18NL273—G18NL280	Heterotic group 1 (N3)
	G16NL677—G16NL813; G16NL815—GL16NL850; GL16NL852—GL16NL921; G17NL373—G17NL467; G17NL469—G17NL484; G17NL486—G17NL664; G18NL665—G18NL676	Heterotic group 2 (SC)

## Genotyping of inbred lines

### Sample collection

The lines were field-established at Rattray Arnold Research Station (RARS) in Zimbabwe. RARS is situated at Longitude 31°12′ 41.35″ E, Latitude 17°40′ 20.07″ S, at an altitude of 1360 metres above sea level. The climate is sub-tropical, with average monthly temperatures ranging from 28 to 32°C between November and April. The total annual precipitation received at RARS is 865mm, mostly between November and April. RARS is located in the mid-altitude moist environments, which are the primary maize-growing areas in Southern Africa. Ten kernel samples were collected for each line. The samples were placed in envelopes, which were sealed and accurately labelled. Healthy and disease-free kernels were sampled for genotyping.

### DNA extraction

DNA extraction and SNP genotyping were performed in the Limagrain Laboratory in France following established protocols. Genomic DNA was isolated from maize kernels using the Kompetitive Allele-Specific (KASP) custom method, and genotyping was facilitated by the LGC KASP system (accessible at http://www.lgcgenomics.com). DNA extraction was done from ten kernels per inbred line, with positive controls included in the initial six wells of a 96-well plate. DNA purity and concentration were assessed using a Nanodrop device (Nano Vue Plus), and the DNA was diluted to concentrations ranging from 81 to 188 μg/L. The DNA was then arrayed onto 384 PCR plates and genotyped using 1201 and 1484 SNP KASP markers for the 182 founder lines and 866 derived tropical lines, respectively, covering all ten maize chromosomes. Polymerase Chain Reaction amplification was performed using Kompetitive Allele-Specific PCR (KASP) primers, generating sufficient DNA for genotyping. The KASP system facilitated the genotyping process, with scanning conducted using Pheraster SNP software and processing carried out using KlusterCaller software (http://www.lgcgenomics.com).

## Data analysis

### Analysis of molecular variance

Analysis of molecular variance (AMOVA) was conducted using GenAlEx 6.51b2 software [32], following the protocol outlined by [32] to partition genetic variations among and within populations attributable to SNPs.

### Characterization of SNP markers and inbred lines

SNP markers with poor amplification, uncertain allele identification, or excessive missing data (>10%) were excluded, as were markers with a minor allele frequency of less than 5%. Inbred lines with the assosciated data missing above 10% were removed from the analysis. After filtering, 1201 SNP markers (S1 Table in S1 File) were used to genotype the 182 founder lines and 1484 markers (S2 Table in S1 File), were used to genotype the 866 derived lines. The inbred lines were genotyped using KlusterCaller software (http://www.lgcgenomics.com) to detect single nucleotide polymorphisms (SNPs) and insertions/deletions (indels), enabling a thorough evaluation of genetic variation. The DARwin6 software [33] was used to identify unique clustering patterns and structure, and generated dendrograms and phylogenetic trees to visualize the relationships between populations.

The 1201 and 1484 markers selected were distributed among the 10 maize chromosomes for the 182 founder and 866 derived lines, respectively. The number of markers per chromosome varied from 59 to 215 in the founder lines and 76 to 231 in the derived lines. The markers were spaced at regular intervals along each chromosome, based on genetic distance expressed in centimorgan (cM). This arrangement ensured full coverage and prevented the clustering of markers, giving a complete overview of the entire genome (Table 2).

**Table 2:** Positioning of the 1201 and 1484 SNP markers on the maize chromosomes using linkage analysis.

Chromosome	Number of markers	Position range (Centimorgans)	Chromosome	Number of markers	Position range (Centimorgans)
1	212	16.06–617.22	1	231	(-29.91) - 619.29
2	114	1.21–443.73	2	173	(-29.69) - 444.94
3	114	(-5.18) - 520.78	3	189	(-5.00) - 521.05
4	84	2.82–378.74	4	151	(-16.32) - 385.80
5	165	5.23–403.51	5	141	(-2.89) - 413.67)
6	105	(-4.28) - 373.85	6	114	(-13.85) - 376.33
7	70	(-44.27) - 425.09	7	135	(-57.04) - 425.09
8	215	(-31.87) - 382.75	8	141	(-34.41) - 362.26
9	59	6.44–350.25	9	130	(-17.33) - 355.45
10	63	1.30–232.45	10	76	(-13.50) - 242.23
	1201			1484	

Genetic parameters were computed, including major allele frequency (MAF), genetic distance (GD), polymorphic information content (PIC) and heterozygosity (He) using Power-Marker (version 3.2.5) statistical software [34]. GD represents the likelihood of two randomly chosen individuals being different at a specific locus, measuring expected heterozygosity [35]. Gene diversity (GD) was calculated as follows [36]:

$$GD=1-\sum_{u=1}^{k}{{\tilde{P}}^{2}}_{u}$$

where

*k =* number of alleles,

${{\tilde{P}}^{2}}_{u}$ = frequency of the marker allele.

Polymorphic information content (PIC) values estimate a marker’s discriminating power by considering the number of alleles. The formula for calculating the PIC was as follows [36]:

$$PIC=1-\sum_{u=1}^{k}{{\tilde{P}}^{2}}_{u}-\sum_{u=1}^{k-1}\sum_{v=u+1}^{k}2{{\tilde{P}}^{2}}_{u}{{\tilde{P}}^{2}}_{V}$$

where:

${{\tilde{P}}^{2}}_{u}$ = frequency of the marker allele,

*k =* number of alleles,

${{\tilde{P}}^{2}}_{u}$ = frequency of the marker allele,

*P*^*2*^_*u*_ = frequency of the *u*^th^ marker,

${{\tilde{P}}^{2}}_{V}$ = frequency of the *v*^th^ marker.

The PIC values were categorized as highly informative (PIC value of the marker >0.50), (ii), moderately informative (0.25 to 0.50) or slightly informative (<0.25) [34].

### Marker call rate

The marker call rate was calculated by determining the proportion of successful genotyping calls for each marker across all samples.

Marker call rate = (Number of successful genotyping calls / Total number of samples) x 100

Where:

- Number of successful genotyping calls = the number of samples for which a genotype was successfully called for a given marker.
- Total number of samples = the total number of samples genotyped for a given marker.

### Population structure analysis

Genetic data from 1201 and 1484 SNP markers were analyzed using the admixture model-based clustering method in STRUCTURE 2.3.4 software [37] to infer the population structure of 182 founder lines and 866 derived lines, respectively. The analysis settings included a burn-in period of 20,000 iterations, a Markov chain Monte Carlo (MCMC) simulation length of 100,000, and six independent runs for each K value (ranging from 1 to 7) for the 182 founder lines and ten independent runs for each K value (ranging from 1 to 11) for the 866 derived lines. The optimal number of populations (K) was estimated using the Evanno et al. (2005) method, as implemented in the online STRUCTURE Harvester tool [37].

### Cluster analysis

The genetic relationships among the inbred lines were discerned using DARwin software [33]. The neighbor-joining method (NJ) [38], was used to construct phylogenetic trees with 500 bootstraps using the 1201 SNP marker data for the 182 founder lines and 1484 SNP marker data for the 866 derived lines. Continuous dissimilarity indices were generated using the standard Euclidean similarity test using the formula described by Perrier and Jacquemoud-Collet [33], enabling the construction of dendrograms that illustrate the genetic distances between the lines:

$$d_{ij}=\sqrt{\sum_{1}^{k}{\left(X_{ij}-X_{jk}\right)}^{2}}$$

Where; d_ji_ is the similarity between units *i* and *j; Xij*, X_*jk*_ are values for variable *k* for units *I* and *j*,

X represents the global mean, and *k* indicates the number of variables. The output from DARwin was imported into FigTree version 1.4.3 [39]. software to construct the final phylogenetic trees.

## Results

### Summary statistics for the 182 founder and 866 derived lines

**Fig 1:**
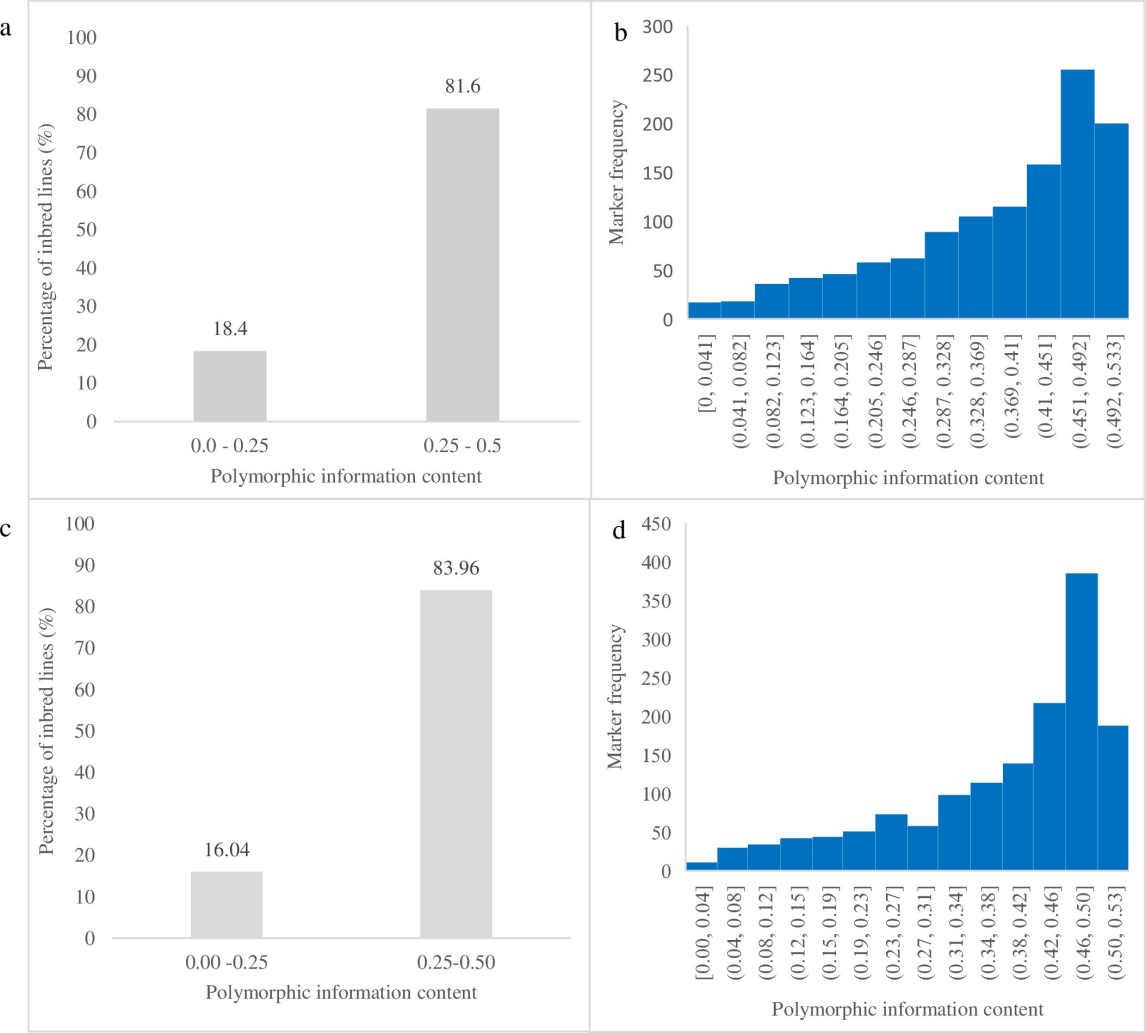
Frequency distribution and marker frequency of 182 parental inbred lines (a and b) and 866 derived inbred lines (c and d) calculated using 1201 and 1484 SNP markers, respectively, based on polymorphic information content.

Table 3 presents the genetic diversity parameters based on SNP markers for the 182 founder and 866 derived lines. The observed heterozygosity for the 182 founder lines was 0.08, ranging from 0.01 to 0.24. Gene diversity ranged from 0.00 to 0.44, with a mean of 0.25, indicating a moderate level of genetic variation. The mean major allele frequencies was 0.85, ranging from 0.50 to 0.99. The polymorphic information content (PIC) ranged from 0.00 to 0.50, with a mean of 0.37. Most markers (82%) were moderately informative (PIC ≥ 0.25), while 18% were slightly informative (PIC < 0.25) (Fig 1). Minor allele frequencies ranged from 0.10 to 0.50, with a mean of 0.28. The marker call rate was high, with a mean of 89.99%, varying from 85.00% to 95.00%.

**Table 3:** Genetic diversity parameters for 182 founder and 866 derived inbred lines of maize calculated using 1201 and 1484 SNP markers, respectively.

Statistics/genetic parameters	MaF	He	Ho	PIC	MAF	Call Rate
	Founder lines					
Minimum	0.50	0.00	0.00	0.00	0.10	85.00
Maximum	0.99	0.44	0.24	0.50	0.50	95.00
Mean	0.85	0.25	0.08	0.37	0.28	89.99
	Derived lines					
Minimum	0.50	0.00	0.00	0.00	0.08	37.22
Maximum	0.99	0.34	0.21	0.50	0.50	100.00
Mean	0.82	0.13	0.08	0.41	0.30	96.64

MaF = Major allele frequency, He = Gene diversity, Ho = Observed heterozygosity, PIC = Polymorphism information content, MAF = Minor allele frequency.

The major allele frequencies based on the 1,484 SNP markers ranged from 0.50 to 0.99, with a mean of 0.82. Gene diversity ranged from 0.00 to 0.34, with a mean of 0.25, while observed heterozygosity averaged 0.08, ranging from 0.00 to 0.21. The PIC values ranged from 0.00 to 0.50, with a mean of 0.41. Most markers (84%) were moderately informative (PIC ≥ 0.25), while 16% were slightly informative (PIC < 0.25) (Fig 1). The minor allele frequencies ranged from 0.08 to 0.50, with a mean of 0.30. The marker call rate ranged from 37.22% to 100%, with a mean of 96.64%, indicating a high genotyping success rate.

### Analysis of molecular variance

Analysis of molecular variance (AMOVA) revealed significant genetic differences (P ≤ 0.001) among and within populations (Table 4). Most genetic variation was attributed to within-population variation, accounting for 97% and 88.38% of the total variation in the founder and derived lines, respectively (Table 4).

**Table 4:** Summary of analysis of molecular variance comparing among and within maize populations of 182 founder inbred lines and 866 derived inbred lines based on 1201 and 1484 SNP markers, respectively.

Founder inbred lines					
Source of variation	Degrees of Freedom	Sum of Squares	Estimated Variance	Percentage of Variation	F Statistics
Among Pops	1	344.555	6.062	3	≤ 0.001
Within Pops	180	39464.951	219.25	97	≤ 0.001
Total	181	39809.505	225.312	100	
Derived inbred lines					
Source of variation	Degrees of Freedom	Sum of Squares	Estimated Variance	Percentage of Variation	F Statistics
Among Pops	2	6568.76	22.98	11.62	≤ 0.001
Within Pops	864	161234.52	179.84	88.38	≤ 0.001
Total	866	167234.52	202.82	100	

Pops = populations

### Population structure of the germplasm panel

**Fig 2:**
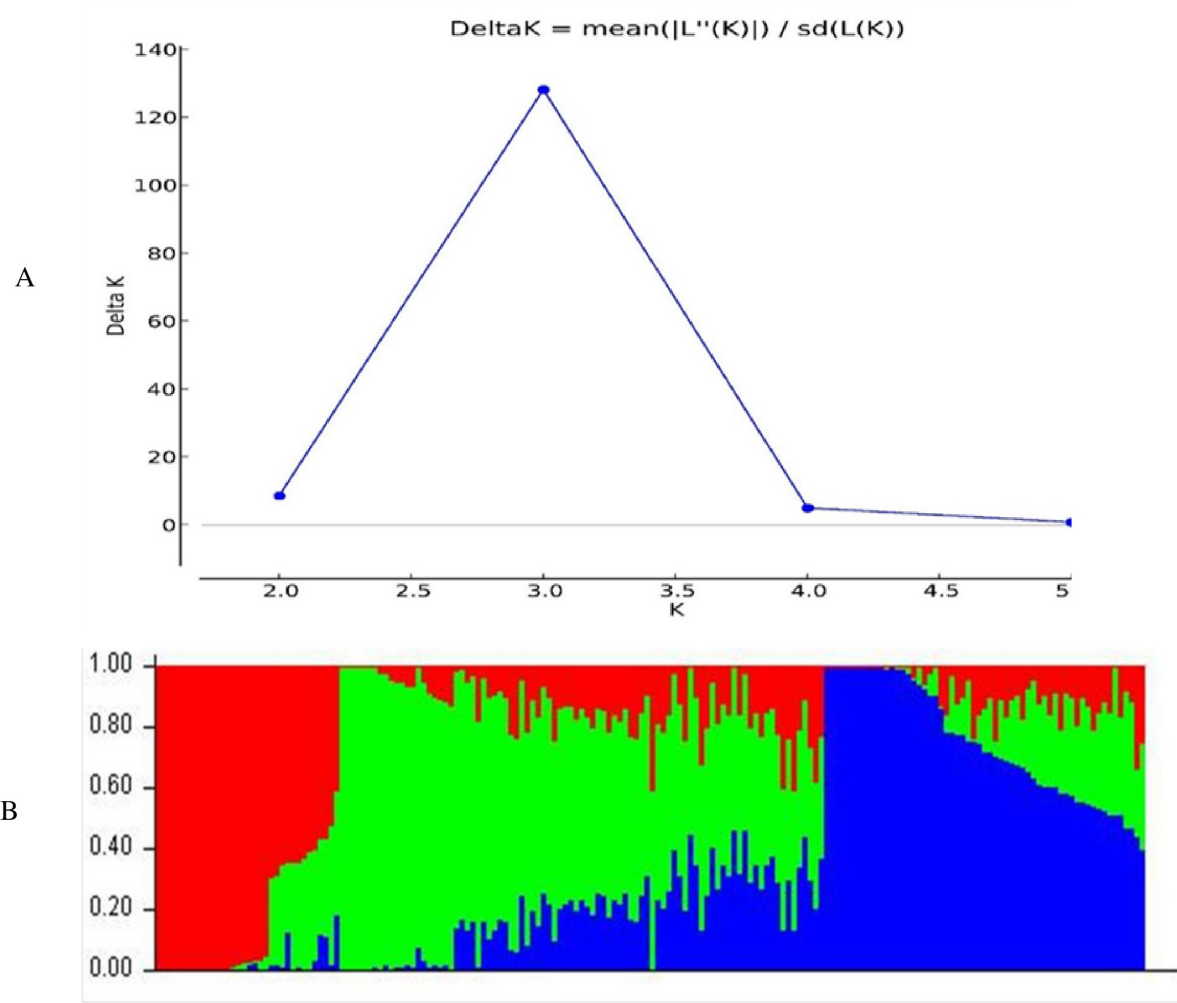
Three sub-populations discerned for the 182 founder inbred lines of maize genotyped using 1201 SNP markers. A—Best Delta K estimation via the Evanno method. B—Estimated population structure of 182 maize inbred lines revealed by 1201 SNP markers for K = 3. Where, I = Sub-population 1, II = Sub-population 2, III = Sub-population 3.

**Fig 3:**
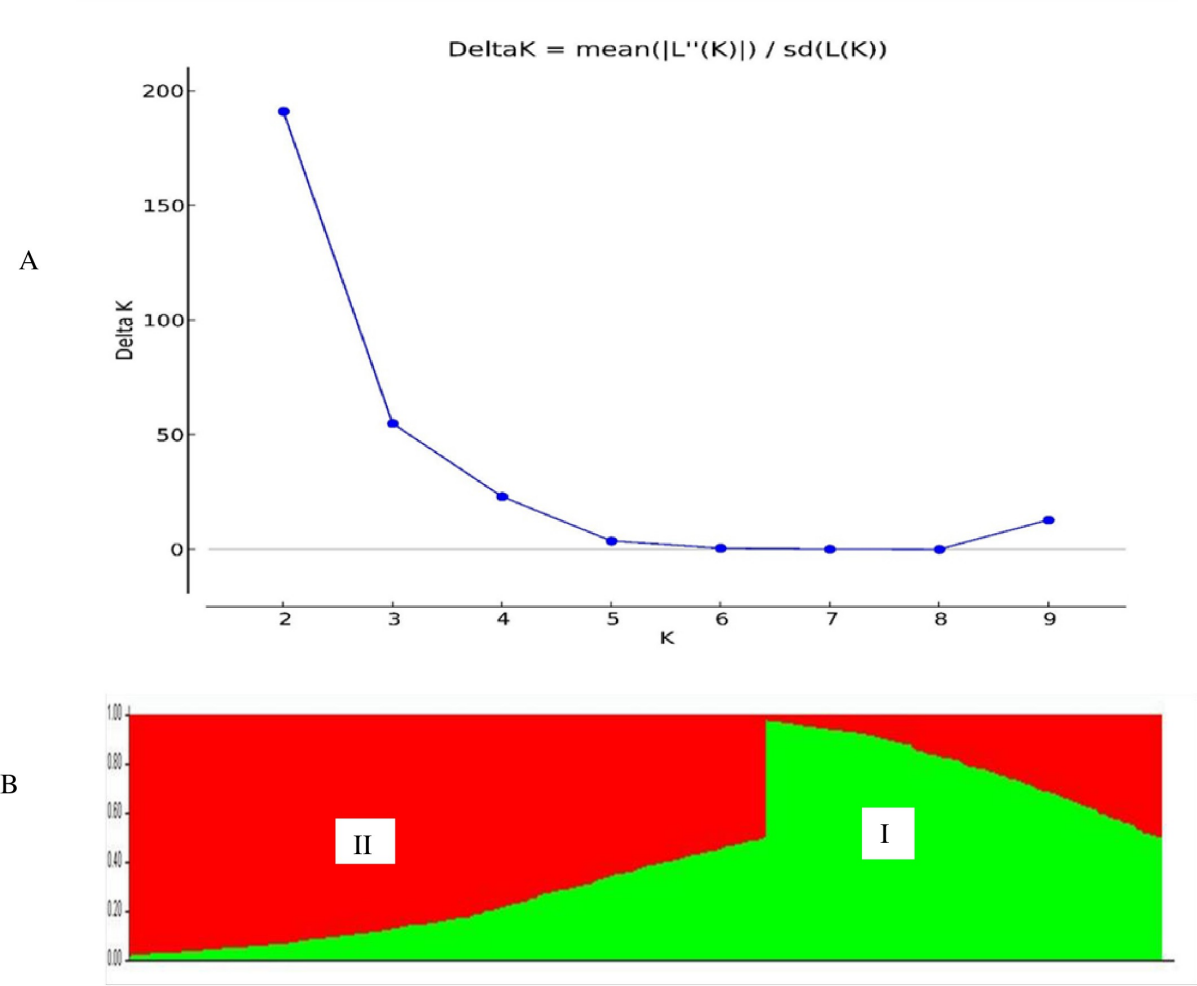
Two sub-populations resolved among the 866 derived inbred lines of maize using 1484 SNP markers. A—Best Delta K estimation via the Evanno method. B—Estimated population structure of 866 maize inbred lines revealed by 1201 SNP markers for K = 2. I = sub-population 1, II = Sub-population 2.

The population structure analysis demarcated the 182 maize lines into three distinct subpopulations (Fig 2), with ΔK peaking at K = 3. The tripartite division captured the underlying genetic diversity, with each cluster varying in size and composition. Subpopulation 1 consisted of 38 lines, Subpopulation 2 had 100 lines, and Subpopulation 3 had 44 lines (S4 Table in S1 File). Further, the 866 maize lines were partitioned into two subpopulations (Fig 3), with the highest ΔK value at K = 2, indicating a bipartite division. The two clusters, identified by the highest median log-probability values (Ln(Pr(Data))), differed in size and composition, with cluster 1 composed of 328 lines and cluster 2 having 538 lines (S5 Table in S1 File).

## Cluster analysis

### Genetic distance and cluster analysis of 182 founder inbred lines

Genetic distance estimates based on SNP markers among the 182 maize inbred lines revealed variable genetic diversity ranging from 0.006 (16AG16785 vs 16AG16786) to 0.435 (RGS-PL33 vs RGS-PL44) (S6 Table in S1 File). The mean genetic distance for all pairwise comparisons was 0.25, indicating moderate genetic diversity among the lines. Low genetic distances were detected between several pairs of inbred lines, including 16AG16786 and 16AG16785 (0.006), 16AG16801 and 16AG16802 (0.013), and RGS-PL17 and RGS-PL55 (0.021), suggesting a high degree of genetic similarity between these lines. In contrast, high genetic distances were estimated between RGS-PL33 and RGS-PL44 (0.435), 15AG152 and RGS-PL44 (0.432), indicating a more distant genetic relationship.

**Fig 4:**
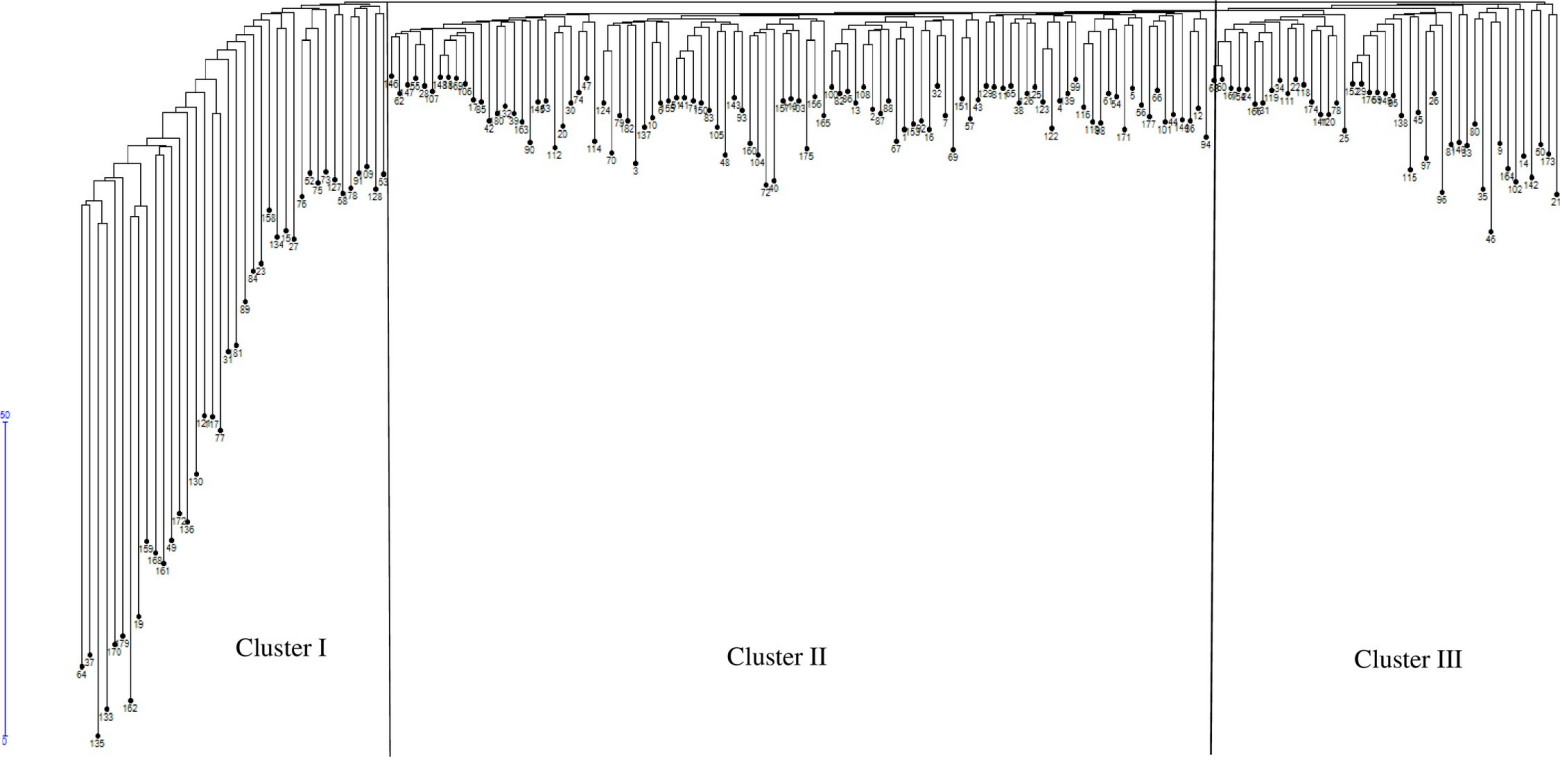
Cluster diagram showing the relationships between 182 inbred lines based on 1201 SNP markers. See S3 Table in S1 File for codes of genotypes.

Genetic grouping based on population structure analysis confirmed the results of the cluster diagram that resolved the 182 genotyped inbred lines into three major clusters (Fig 4). Each cluster was partitioned into sub-clusters, with Cluster II being the largest (comprising 55% of parental inbred lines), followed by Cluster III (24% of inbred lines), and Cluster I (21% of inbred lines). These clusters corresponded to sub-populations 1 (red), 2 (green), and 3 (blue) from the structure analysis, respectively. The phylogenetic tree provided a visual representation of the genetic relationships among the inbred lines, supporting the findings of the population structure analysis.

### Genetic distances and cluster analysis of 866 derived inbred lines

**Fig 5:**
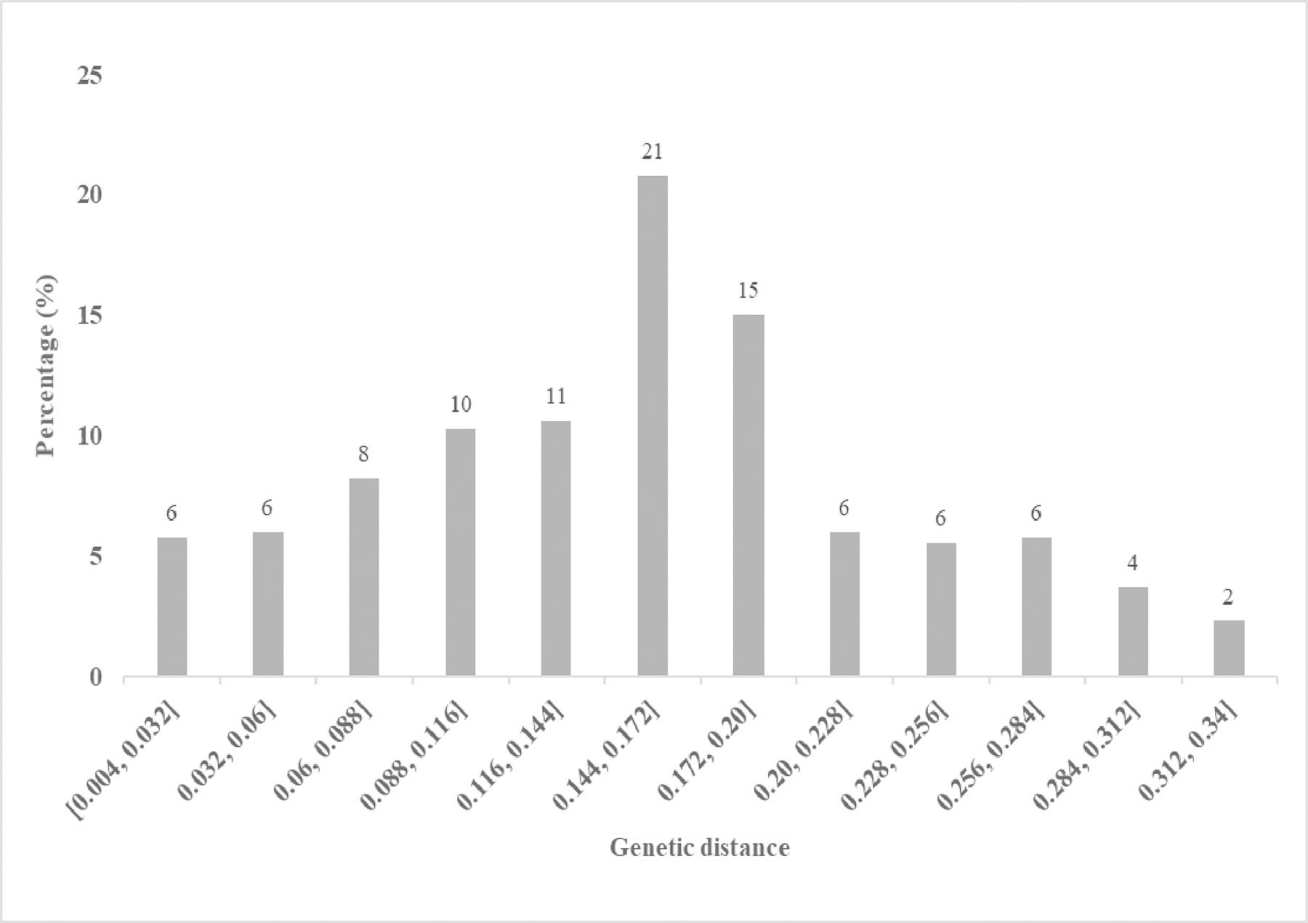
The distribution of pairwise genetic distance calculated for 866 maize inbred lines genotyped with 1484 SNPs.

**Fig 6:**
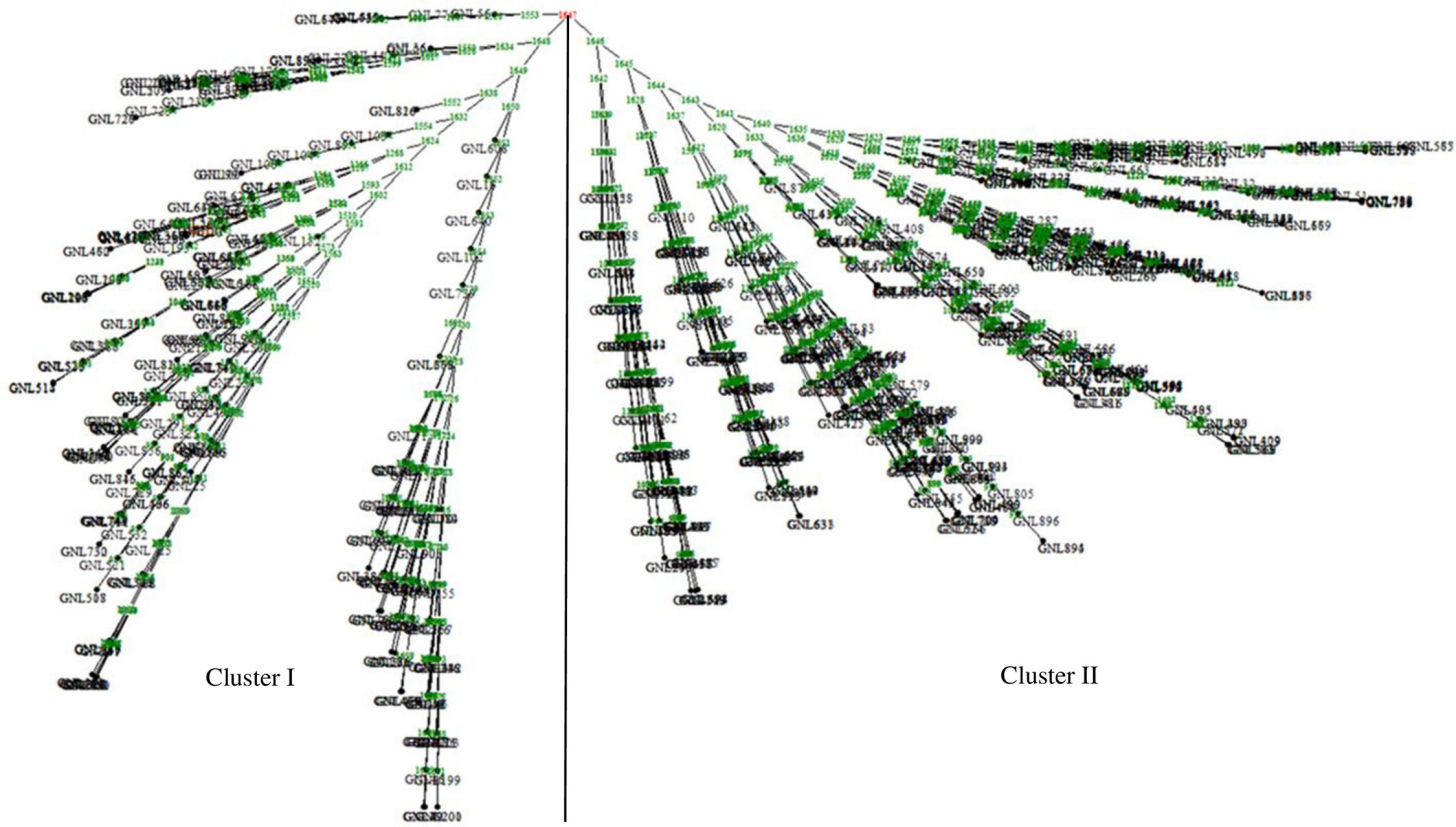
Phylogenetic relationships among 866 derived inbred lines, as inferred from 1484 SNP markers revealing distinct clusters and branches. See S5 Table in S1 File for codes of genotypes.

The genetic distance between pairwise comparisons based on 1484 SNP markers for the 866 derived lines ranged from 0.004 to 0.336 (S7 Table in S1 File), with a mean genetic distance of 0.14. Most lines (77%) had genetic distances ranging from 0.004 to 0.20, while 33% had distances above 0.20, ranging from 0.20 to 0.34 (Fig 5). The lowest genetic distance (0.004) was recorded between the inbred lines G17NL211 and G17NL210. Other pairs of lines with low genetic distances included G17NL473 and G17NL472 (0.005), G17NL194 and G17NL472 (0.008), G16NL854 and G16NL857 (0.009), G17NL602 and G17NL603 (0.011), and G16NL919 and G16NL920 (0.015). Conversely, the highest genetic distance was recorded between lines G15NL337 and G15NL312 (0.336), followed by G15NL349 and G15NL310 (0.31), G15NL303 and G15NL357 (0.303), G15NL327 and G15NL353 (0.301), G15NL292 and G15NL284 (0.299), and G15NL355 and G15NL301 (0.298). Cluster analysis of the 866 derived lines based on SNP marker genetic distance estimates grouped the lines into two distinct clusters (Fig 6), agreeing with the structure analysis (Fig 3). Each cluster was partitioned into sub-clusters. Cluster I, corresponding to Sub-population 1 from the population structure analysis (highlighted green), was designated as heterotic group 1 and composed 328 lines (37.88%). Cluster II, corresponding to Sub-population 2 (highlighted red), was designated heterotic group 2 with 538 lines (62.12%).

## Discussion

Knowledge of genetic diversity, structure, and genetic relationships among elite inbred line populations of maize is vital for selecting genetically diverse and complementary lines for hybrid breeding and enhancing heterotic groups. Hybrid varieties are the best yielders, buffer biotic and abiotic stresses and achieve economic returns. Best cross combinations are selected based on maximum heterosis, which requires the development of homozygous elite lines and a subsequent assortment of contrasting heterotic patterns. Inbred lines with shared ancestry may lead to heterogeneous populations, limiting hybrid vigour. Inbred lines can be crossed, and a new generation of derived lines can be selected that are genetically complementary and distinct from other heterotic populations [20, 27].

High-throughput genotyping methods effectively determine and delineate the genetic relationships among inbred populations. Diagnostic molecular markers provide a high level of accuracy in identifying the genetic constitution of individuals to guide hybrid breeding [13, 16, 40]. The SNP markers are widely used to assess genetic diversity and relationships [28, 41], select breeding parents [42, 43], and identify novel genes linked to economically important traits [44, 45]. Also, SNPS are the genetic markers of choice due to several advantages, including their low cost per data point, widespread presence in the genome, specific location at genetic loci, co-dominance, and lower rates of genotyping errors [26, 27].

The present study evaluated the extent of genetic diversity, population structure, and genetic lineage of a maize germplasm panel consisting of 182 founder lines and 866 derived inbred lines using 1201 and 1484 SNP markers, respectively. The analysis revealed insights into allele frequency, which is crucial for understanding the extent of genetic variation among populations [46]. The major allele frequency varied from 0.50 to 0.99 (Table 3), revealing adequate genetic variation among the test populations for selection and potential for marker-assisted breeding programs. The mean MaF values for the 1201 and 1484 SNP markers were 0.82 and 0.85, respectively, indicating a high frequency of dominant alleles. The mean MAFs were 0.28 and 0.30 for the 1201 and 1484 markers, respectively (Table 3), indicating a moderate distribution of alleles among the inbred lines. This was consistent with previous reports by [15, 47], but lower than those reported by [48, 49]. Minor alleles indicate genetic variations of a specific gene or genetic marker crucial for preserving diversity within a population [15, 23, 50].

The assessed tropical elite maize lines had an average observed heterozygosity of 0.24 (Table 3), indicating a high degree of homozygosity and genetic stability. However, since inbred lines with heterozygosity > 5% are considered impure, some of the lines in this study may need additional selfing to reach the desired level of genetic purity. These findings align with [20], who reported lower heterozygosity in early-maturing tropical maize inbred lines using SNP analysis. Low mean heterozygosity values of 0.20 were also reported by [51, 52]. The polymorphism information content predicts the relevance of a genetic marker for linkage analysis [31, 53]. In this study, the mean PIC values for the 1201 and 1484 markers were 0.37 and 0.41, respectively (Table 3), indicating that the SNP markers used were moderately informative. The moderate genetic polymorphism detected in this study is in line with the expected characteristics of bi-allelic SNP markers, which are limited to a maximum PIC value of 0.5. Nevertheless, the PIC values obtained in this study are still informative and can be used to evaluate the genetic diversity and relationships among the tropical elite maize lines [54, 55], and the lower mutation rates of SNPs compared to other genetic markers [56]. SNP markers are more precise in genetic analysis. However, SNPs show lower PIC values than other markers, such as SSRs [57].

Genetic distance (GD) measures the genetic difference or dissimilarity among genotypes in a population and can be used to infer their genetic relationships [20, 24, 49]. The present results showed considerable genetic variability among the inbred lines (Table 3). Genetic distances among the founder lines ranged from 0.006 to 0.44, with a mean of 0.25. The values suggest high genetic diversity and differentiation levels in the founder parental lines. The genetic distance values detected in the founder lines agree with previous studies in maize [20, 58]. However, these values were lower than those reported by other researchers [13, 28], who reported average genetic distance values of 0.32, as well as [59, 60], who reported values of 0.37. The derived elite lines showed moderate genetic distances (mean of 0.13), indicating a reasonable degree of genetic variation among individuals while sharing significant genetic similarity (Table 3).

The SNP panels used in this study identified adequate genetic polymorphisms among and within the inbred line populations (Table 4). The low moderate diversity detected within the founder and derived inbred lines were attributable to genetic drift, founding effects, artificial selection, genetic recombination and linkage disequilibrium. A higher degree of genetic diversity at 97% that accounted for the within-population genetic variation was computed for the founder inbred line populations. In contrast, the variation among populations was low, at 3%, suggesting high gene flow due to outcrossing, genetic drift, founding effect and artificial selection. In the derived lines, among-population variation was 11.62%, while the within-population variation was higher at 88.38% (p≤0.001) signifying that the majority of genetic variation was partitioned within the population, suggesting a high level of genetic heterogeneity within the population. The high genetic diversity within the population exhibited by both elite sets of lines is a valuable asset for breeding and conservation purposes.

Population structure analysis determines the genetic ancestry of inbred lines [61, 62]. The genetic analysis using the SNP analysis delineated three subpopulations (K = 3) for the founder lines (Fig 2). The SNP markers effectively categorized the inbred lines into heterotic groups based on their source populations, grouping individuals with similar genetic backgrounds into the same subpopulations. The identified population groups guide breeding programs to select parental lines. The current results agree with [20], who reported three subpopulations among 94 early maturing tropical maize inbred lines using SNP markers. Similarly, based on the neighbour-joining cluster analysis, the dendrogram (Fig 4) allocated the founder inbred lines into three genetic groups. Population analysis involving the 866 derived lines revealed two sub-populations (K = 2) (Fig 3). The inbred lines were assigned into heterotic groups based on similarity of ancestry and selection history. The genetic clustering based on the neighbour-joining cluster analysis supported the findings based on phylogenetic analysis. The grouping of the derived lines into two sub-populations is consistent with findings by [13], who identified two groups (K = 2) among 770 maize inbred lines using SNPs.

## Conclusion

The present study assessed the genetic diversity and population structure comprising 182 founder lines and 866 derived inbred lines using diagnostic SNP markers and identified genetically unique lines for hybrid breeding. Higher genetic variations, at 97% and 88.38%, were attributed to within populations in the founder and derived lines, in that order. Population structure analysis identified three distinct genetic groups among founder lines and two among derived lines. Based on pairwise genetic comparison, the following founder and derived inbred lines were selected: G15NL337 and G15NL312 (Cluster 1), 15ARG152 and RGS-PL44 (Cluster 2), RGS-PL44 and 15ARG149 (Cluster 2), and RGS-PL33 and RGS-PL44 (Cluster 2), respectively. The selected lines are genetically distinct and recommended for marker-assisted hybrid maize breeding to exploit the frequency of beneficial alleles. The study identified novel genetically distant founder lines (i.e., 15ARG152 and RGS-PL44 and RGS-PL44 and 15ARG149) and derived lines (G15NL337 and G15NL312). The SNP markers identified with high polymorphism information content are valuable in genomic selection and genetic analysis and breeding. The core findings of the study are valuable references for maize breeding programs in Africa when using the current and related tropical-adapted populations.

## Supporting information

S1 File (ZIP)
