# Correction: Genetic diversity and population structure analyses of tropical maize inbred lines using Single Nucleotide Polymorphism markers

**DOI:** 10.1371/journal.pone.0341231

**Published:** 2026-01-20

**Authors:** Rodreck Gunundu, Hussein Shimelis, Seltene Abady Tesfamariam

There are errors in the author affiliations. The correct affiliations are as follows:

Rodreck Gunundu^1,2^, Hussein Shimelis^1^, Seltene Abady Tesfamariam^1^

**1** African Centre for Crop Improvement (ACCI), College of Agriculture, Engineering and Science (CAES), University of KwaZulu-Natal, Scottsville, Pietermaritzburg, South Africa, **2** Seed Co, Rattray Arnold Research Station, Harare, Zimbabwe
