# Supplementary material for: Genetic diversity and population structure analyses of tropical maize inbred lines using Single Nucleotide Polymorphism markers
Source: PLoS One. 2025 Jan 24;20(1):e0315463. doi: 10.1371/journal.pone.0315463 (PMC11760008; doi:10.1371/journal.pone.0315463)
Supplement: S1 File — (ZIP) [file pone.0315463.s001.zip › Supplementary Table 1.docx]

Supplementary Table 1. SNP markers used for genotyping 182 founder maize inbred lines.

| CHR | SNP ID |
| --- | --- |
| 1 | ASSAY10208_00415, ASSAY1173_00197, ASSAY1290_00096, ASSAY1742_00143, ASSAY1751_00401, ASSAY1796_00123, ASSAY1796_00360, ASSAY1796_00457, ASSAY1822_00212, ASSAY1941_00169, ASSAY1994_00352, ASSAY2001_00126, ASSAY2001_00205, ASSAY2110_00115, ASSAY2165_00126, ASSAY2192_00127, ASSAY2214_00106, ASSAY2271_00573, ASSAY2483_00144, ASSAY2512_00251, ASSAY2654_00092, ASSAY2654_00154, ASSAY2654_00311, ASSAY2731_00185, ASSAY2757_00158, ASSAY2769_00153, ASSAY2803_00283, ASSAY2831_00093, ASSAY2831_00252, ASSAY2831_00371, ASSAY2911_00359, ASSAY2927_00166, ASSAY3117_00033, ASSAY3127_00216, ASSAY3135_00148, ASSAY3190_00322, ASSAY327_00151, ASSAY3292_00365, ASSAY3403_00064, ASSAY552_00279, ASSAY565_00217, ASSAY5847_00205, ASSAY6798_00109, ASSAY6817_00814, ASSAY731_00266, ASSAY748_00301, ASSAY777_00159, ASSAY8459_00493, ASSAY8459_01125, ASSAY8471_00031, ASSAY8517_00198, ASSAY8790_00455, ASSAY8795_00060, ASSAY8925_00294, ASSAY8926_00468, ASSAY925_00067, ASSAY931_00121, ASSAY948_00179, ASSAY955_00148, BGA00036A1_00150, BGA00036A1_00580, BGA00085F4R7_00215, BGA00131F2R2A1_00351, BGA00140F1R1_00647, EPG00057F3R3_00140, EPG00057F3R3_00337, EPG00057F3R3_00614, EPG00057F3R3_00800, KWS00021F3R3_00417, LIM00048_00345, LIM00049F5R5_00364, LIM00132_00027, LIM00133_00152, PUT-163A-31997542-2011, PUT-163A-60344363-2515, PUT-163A-60350073-2631, PUT-163A-71305340-3082, PUT-163A-76012177-3730, PUT-163A-86470789-4503, PUT-163A-88747038-4526, PZA00468.8, PZA02359.10, PZA02957.5, PZA03557.1, PZB01915.1, PZE-101009163, PZE-101023852, PZE-101024808, PZE-101033622, PZE-101035737, PZE-101039207, PZE-101048857, PZE-101049091, PZE-101057118, PZE-101068816, PZE-101160171, PZE-101160748, PZE-101162399, PZE-101163301, PZE-101163849, PZE-101163883, PZE-10116815, PZE-101197298, PZE-101198605, PZE-101198781, PZE-101199832, PZE-101200344, PZE-101201219, PZE-101201492, PZE-101203040, PZE-101203924, PZE-101205031, PZE-101205714, PZE-101206027, PZE-101206252, PZE-101207067, PZE-101207166, PZE-101207892, PZE-101208157, PZE-101208921, PZE-101209157, PZE-101210110, PZE-101210734, PZE-101211692, PZE-10121411, PZE-101214804, PZE-101215143, PZE-101215699, PZE-101216104, PZE-101217024, PZE-101218183, PZE-101221171, PZE-101222284, PZE-101224367, PZE-101225113, PZE-101225132, PZE-101226725, PZE-101227145, PZE-101228665, PZE-101229195, PZE-101229632, PZE-101230194, PZE-101230537, PZE-101232138, PZE-101233856, PZE-101234638, PZE-101236600, PZE-101236759, PZE-101237912, PZE-101238691, PZE-101247063, PZE-101249551, PZE-101249703, PZE-101251539, PZE-101252431, PZE-101256110, PZE-101256470, RAG00037F1R1_00354, RAG00037F1R1_00408, RAG00037F1R1_00562, RAG00119F4R5_00426, RAG00119F4R5_00436, RAG00119F4R5_00507, RAG00148F1R2_00334, RAG00194F2R2_00540, SYN14016, SYN15630, SYN16862, SYN16865, SYN2024, SYN21366, SYN21642, SYN27356, SYN27374, SYN297, SYN38906, SYN38939, SYN8233, SYN8236, ZM000417_0480, ZM000434_1802, ZM001195_0628, ZM001629_0989, ZM001719_0119, ZM002382_0505, ZM002383_0403, ZM002689_0540, ZM002776_0860, ZM002985_0179, ZM003044_1290, ZM003900_0697, ZM004071_0670, ZM004694_0326, ZM005800_0217, ZM006103_0363, ZM006103_0446, ZM006288_1134, ZM006877_0585, ZM007810_0794, ZM008178_0127, ZM008698_0833, ZM008740_0593, ZM009424_0548, ZM009680_0375, ZM010129_0558, ZM010322_0169, ZM010458_0571, ZM010563_0342, ZM010766_1155, ZM011281_0570, ZM011857_0402, ZM012324-0461 |
| 2 | ASSAY10122_00805 ASSAY1044_00085 ASSAY1114_00580 ASSAY1114_00781 ASSAY1119_00037 ASSAY1247_00558 ASSAY1247_00728  ASSAY1733_00295 ASSAY1758_00513 ASSAY1914_00276 ASSAY1938_00167 ASSAY1938_00230 ASSAY2043_00246 ASSAY2071_00243  ASSAY2073_00097 ASSAY2073_00173 ASSAY2147_00289 ASSAY2155_00070 ASSAY2190_00350 ASSAY2213_00210 ASSAY2265_00173  ASSAY2342_00148 ASSAY2393_00257 ASSAY2570_00292 ASSAY2575_00306 ASSAY2631_00244 ASSAY2794_00355 ASSAY2977_00175  ASSAY2977_00251 ASSAY3002_00282 ASSAY3011_00156 ASSAY3189_00146 ASSAY3204_00230 ASSAY3321_00088 ASSAY3372_00119  ASSAY464_00037 ASSAY533_00323 ASSAY533_00323 ASSAY560_00580 ASSAY591_00467 ASSAY5927_00412 ASSAY5987_00294  ASSAY6016_00056 ASSAY715_00153 ASSAY745_00164 ASSAY8505_00418 ASSAY8505_00866 ASSAY8699_00172 ASSAY8699_00692 ASSAY872_00169 ASSAY8869_00366 ASSAY8908_00230 ASSAY8922_00092 ASSAY8924_00420 BGA00027F5R5_00436 BGA00118F4R4_00053  BGA00212F3R3_00238 BGA00234F2R2_00073 BGA00306F1R1_00480 BGA00370F1R1_00095 EPG00113_00630 EPG00139F2R2_00446 EPG00210F2R2_00262 LIM00097_00903 LIM00151F199R199_00376 PZA02727.1 PZE-102063478 PZE-102067230 PZE-102069040 PZE-102069195  PZE-102070420 PZE-102084771 PZE-102098807 PZE-102183145 PZE-102184919 PZE-102185011 PZE-102185564 PZE-102185952 PZE-102191279  PZE-102192375 PZE-102192647 PZE-102193034 PZE-102194180 PZE-110090076 RAG00169F2R2_00214 RAG00210F2R2_00088 SYN14631 SYN18301 SYN20670 SYN28307 SYN34400 ZM000641_0697 ZM001125_1218 ZM001293_0732 ZM001525_0541 ZM001679_0624 ZM002806_0674 ZM003338_0691 ZM003429_0308 ZM004340_0418 ZM005597_1446 ZM005925_0931 ZM006229_0205 ZM006881_0736 ZM006881_1075 ZM007140_0341 ZM007489_0332 ZM007489_0517 ZM007621_0429 ZM007621_0513 ZM008336_0716 ZM009029_0412 ZM009592_0683ZM010663_0466 |
| 3 | ASSAY1041_00081 ASSAY1155_00094 ASSAY1491_00173 ASSAY1655_00046 ASSAY1709_00246 ASSAY1727_00379 ASSAY1790_00152  ASSAY1790_00238 ASSAY1792_00090 ASSAY1850_00038 ASSAY1862_00241 ASSAY1968_00249 ASSAY2040_00240 ASSAY2209_00431  ASSAY2360_00260 ASSAY2375_00184 ASSAY2375_00199 ASSAY2449_00266 ASSAY2467_00365 ASSAY2480_00293 ASSAY2520_00222  ASSAY2602_00191 ASSAY2762_00058 ASSAY2895_00094 ASSAY2939_00073 ASSAY2947_00197 ASSAY2994_00089 ASSAY3056_00259  ASSAY3151_00129 ASSAY3308_00312 ASSAY3314_00146 ASSAY3382_00159 ASSAY345_02832 ASSAY5844_00439 ASSAY585_00088  ASSAY5861_00269 ASSAY606_00412 ASSAY6474_00916 ASSAY6802_00267 ASSAY817_00190 ASSAY8485_00254 ASSAY8509_00130 ASSAY8509_00643 ASSAY8524_00236 ASSAY8526_00249 ASSAY8704_00418 ASSAY8786_00045 ASSAY8786_00094 ASSAY8786_00290  ASSAY8791_00526 ASSAY8791_00691 ASSAY8791_00725 ASSAY926_00114 ASSAY942_00058 BGA00218F6R6_00295 BGA00369F1R1_00442  BGA00448F1R1_00615 EPG00011F2R2_00189 EPG00011F2R2_00375 EPG00158F1R1_00266 LIM00091_00390 PUT-163A-78113073-4314 PZB00228.3 PZE-103025094 PZE-103034532 PZE-103045772 PZE-103072561 PZE-103076837 PZE-103077185 PZE-103077912 PZE-103078702 PZE-103083512 PZE-103083722 PZE-103084005 PZE-103085573 PZE-103089292 PZE-103090476 PZE-103091693 PZE-103100115 PZE-103102170 QTL3MADSF21R21_00167 QTL3MADSF21R21_00211 RAG00056F1R1_00050 RAG00066F1R1_00497 RAG00089F2R2_00133 RAG00102F1R1_00242 RAG00136F2R2_00398 ZM000452_0355 ZM000515_1189 ZM000583_1178 ZM001101_1649 ZM001140_0258 ZM001683_0381 ZM001754_0273 ZM001839_1234 ZM002328_0630 ZM002911_1254 ZM003785_0433 ZM004333_1181 ZM004456_0637 ZM006120_1056 ZM006442_0209 ZM007184_0534 ZM007594_0420 ZM008693_0358 ZM008759_0264 ZM008789_0123 ZM008829_0535 ZM009167_0320 ZM009707_0213 ZM009949_0423 ZM011057_0368 ZM011243_0160 ZM011785_0468 |
| 4 | ASSAY1185_00104 ASSAY1239_00197 ASSAY1478_00151 ASSAY1575_00216 ASSAY1671_00521 ASSAY1752_00165 ASSAY1766_00265  ASSAY1836_00272 ASSAY1884_00203 ASSAY1947_00152 ASSAY1983_00589 ASSAY2205_00233 ASSAY2266_00235 ASSAY2292_00417  ASSAY2405_00296 ASSAY2405_00395 ASSAY2490_00071 ASSAY2566_00059 ASSAY2628_00297 ASSAY2682_00132 ASSAY2787_00275  ASSAY2791_00512 ASSAY294_00734 ASSAY294_01086 ASSAY2998_00221 ASSAY3119_00494 ASSAY3121_00229 ASSAY3142_00055  ASSAY3149_00105 ASSAY3149_00138 ASSAY3177_00359 ASSAY3407_00142 ASSAY5872_00203 ASSAY5982_00454 ASSAY645_00091 ASSAY8521_00453 ASSAY8529_00502 ASSAY8860_00096 BGA00099F2R2_00340 BGA00099F2R2_00475 BGA00425F1R1_00042 BGA00425F1R1_00394 EPG00128F3R3_00639 KWS00017F1R1_00083 KWS00020F7R7_00170 LIM00021A1_00677 LIM00032F2R2_00435 LIM00057F3R5_00436 LIM00070F1R1_00352 PZE-104073794 PZE-104076811 PZE-104078833 PZE-104079748 PZE-104081085 PZE-104082522  PZE-104083719 PZE-104086265 PZE-104087964 PZE-104089424 PZE-104090186 PZE-104093384 PZE-104094429 PZE-104096416 PZE-104099220  PZE-104113793 PZE-104117537 PZE-104157695 RAG00053A1_03158 RAG00170F1R1_00414 SYN1891 SYN36306 ZM003235_0672 ZM003802_0593  ZM004804_0307 ZM004938_1249 ZM005435_0536 ZM007188_0227 ZM007280_0528 ZM007486_0865 ZM009060_1185 ZM011028_0260  ZM011278_0547 ZM012058_0561 ZM012531-0288 |
| 5 | ASSAY10179_00299 ASSAY1034_00221 ASSAY1079_00139 ASSAY1278_00173 ASSAY1283_00016 ASSAY1283_00276 ASSAY1323_00041  ASSAY1460_00385 ASSAY1481_00174 ASSAY1507_00119 ASSAY1679_00065 ASSAY1679_00233 ASSAY1825_00090 ASSAY1903_00108  ASSAY1943_00172 ASSAY2194_00155 ASSAY2224_00379 ASSAY2236_00266 ASSAY2442_00291 ASSAY2518_00368 ASSAY2623_00253  ASSAY2690_00290 ASSAY2783_00362 ASSAY2846_00159 ASSAY3165_00391 ASSAY3281_00448 ASSAY3281_00537 ASSAY3316_00259  ASSAY3370_00098 ASSAY3391_00366 ASSAY3391_00429 ASSAY514_00169 ASSAY543_00190 ASSAY543_00195 ASSAY545_00248 ASSAY550_00069 ASSAY5925_00055 ASSAY6006_00305 ASSAY677_00139 ASSAY705_00179 ASSAY759_00095 ASSAY851_00163 ASSAY8528_00307 ASSAY8733_00321 ASSAY8733_00475 ASSAY882_00189 ASSAY8976_00390 AVE00038_00142 AVE00055_00213 BGA00008F5R5_00382 BGA00022F7R7_00391 BGA00041A1_00051 BGA00041A1_00716 BGA00041A1_01728 BGA00132F1R1A1_00441 BGA00137A1_00132 BGA00180F1R1_00213 BGA00201F1R1_00428 BGA00469F1R1_00634 EPG00118_00267 EPG00118_00398 LIM00003A1_00132 LIM00003A1_00512 LIM00003A2_00059 LIM00118F236R236_00041 PUT-163A-60350211-2639 PUT-163A-76291488-3952 PUT-163A-78086550-4187 PZA00255.17 PZB00182.1 PZE-105017042 PZE-105020816 PZE-105021941 PZE-105022201 PZE-105022328 PZE-105022450 PZE-105022509 PZE-105022516 PZE-105022751 PZE-105024460 PZE-105024579 PZE-105024985 PZE-105025087 PZE-105025224 PZE-105026164 PZE-105026330 PZE-105027872 PZE-105029444 PZE-105030407 PZE-105031034 PZE-105031086 PZE-105031696 PZE-105031717 PZE-105033399 PZE-105033935 PZE-105042856 PZE-105042963 PZE-105046237 PZE-105046457 PZE-105064517 PZE-105073571 PZE-105073579 PZE-105074639 PZE-105076159 PZE-105078900 PZE-105078909 PZE-105081557 PZE-105082311 PZE-105082431 PZE-105082736 PZE-105083429 PZE-105086726 PZE-105089936 PZE-105090165 PZE-105091062 PZE-105091854 PZE-105092539 PZE-105093999 PZE-105096172 PZE-105097260 PZE-105101487 PZE-105102442 PZE-105103245 PZE-105105756 PZE-105105940 PZE-105106949 PZE-105107897 PZE-105109345 PZE-105111088 PZE-105115780 PZE-105117617 PZE-105118656 PZE-105122814 PZE-105123635 PZE-105126583 RAG00081F3R3_00237 RAG00198F3R4_00526 SYN23466 SYN27136 SYN903 SYN9877 ZM000340_0671 ZM000390_0106 ZM000390_0332 ZM000480_0999 ZM001117_2665 ZM001260_1203 ZM001312_0511 ZM002063_0840 ZM002399_1206 ZM003472_2272 ZM004846_0778 ZM005379_0825 ZM005594_1051 ZM006776_0441 ZM006987_0455 ZM007333_0355 ZM007334_0207 ZM008222_0813 ZM008357_0643 ZM008589_0259 ZM009245_0373 ZM009878_0471 ZM010486_0724 ZM011982_1108 |
| 6 | ASSAY1121_00089 ASSAY1176_00186 ASSAY1414_00933 ASSAY1431_00504 ASSAY1557_00064 ASSAY1569_00169 ASSAY1747_00024  ASSAY1780_00174 ASSAY1821_00182 ASSAY2019_00403 ASSAY2149_00321 ASSAY2288_00756 ASSAY2460_00193 ASSAY2688_00172  ASSAY2887_00179 ASSAY3036_00225 ASSAY3246_00100 ASSAY3246_00169 ASSAY3328_00559 ASSAY3354_00231 ASSAY3356_00059  ASSAY4698_00387 ASSAY470_00254 ASSAY551_00218 ASSAY6507_00641 ASSAY651_00398 ASSAY912_00159 ASSAY984_00356 BGA00114A1_00591 BGA00122F1R1_00051 EPG00065F3R3_00496 EPG00220F1R1_00270 KWS00031F1R1_00359 LIM00134A1_00705  LIM00147F255R255_00200 PUT-163A-149095867-900 PUT-163A-60339740-2390 PUT-163A-88754607-4573 PZE-106000516 PZE-106000855 PZE-106003964 PZE-106004214 PZE-106004598 PZE-106005094 PZE-106007130 PZE-106007950 PZE-106008070 PZE-106008513 PZE-106008760 PZE-106011274 PZE-106013072 PZE-106017319 PZE-106017695 PZE-106017960 PZE-106019566 PZE-106020586 PZE-106022944 PZE-106023074 PZE-106025255 PZE-106026913 PZE-106027037 PZE-106031939 PZE-106035034 PZE-106036132 PZE-106037023 PZE-106037935 PZE-106039805 PZE-106041765 PZE-106042213 PZE-106045060 PZE-106053038 PZE-106053973 PZE-106056051 PZE-106056472 PZE-106056649 PZE-106057131 PZE-106058198 PZE-106058841 PZE-106058984 PZE-106059789 PZE-106060570 PZE-106065562 PZE-106066647 PZE-106072681 PZE-106073001 PZE-106073260 PZE-106073551 PZE-106074780 PZE-106075248 PZE-106079337 RAG00093F2R2_00079 RAG00156F1R1_00251 SYN22590 SYN26886  SYN30854 ZM000270_0816 ZM000410_0464 ZM000960_0819 ZM002026_0226 ZM007813_1059 ZM009406_0679 ZM009685_0442 ZM010559_0680  ZM012949-0478 ZM013982-0467 |
| 7 | ASSAY10216_00198 ASSAY10216_00345 ASSAY1223_00125 ASSAY1315_00053 ASSAY1456_00072 ASSAY1463_00069 ASSAY1519_00068  ASSAY1530_00061 ASSAY1530_00475 ASSAY1612_00117 ASSAY1768_00296 ASSAY1784_00267 ASSAY2158_00076 ASSAY2477_00443  ASSAY3062_00266 ASSAY3231_00263 ASSAY3283_00258 ASSAY3329_00186 ASSAY3331_00086 ASSAY4658_00368 ASSAY532_00155  ASSAY5895_00303 ASSAY5934_00433 ASSAY5968_00269 ASSAY5981_00227 ASSAY640_00070 ASSAY706_00162 ASSAY737_00082  ASSAY753_00099 ASSAY822_00141 ASSAY8523_00512 ASSAY8734_00100 ASSAY8734_01030 ASSAY8792_00171 ASSAY8857_00501  ASSAY8857_00917 ASSAY8896_00155 ASSAY8899_00146 ASSAY8918_00231 ASSAY986_00302 BGA00181A1_00649 BGA00213F2R2_00291  BGA00244F7R7_00145 EPG00154F2R2_00046 PZE-107085004 RAG00084F2R2_00552 RAG00135F1R1_00376 SYN23679 ZM000443_0535  ZM000468_0476 ZM000654_0808 ZM000654_0964 ZM000705_0554 ZM000843_1652 ZM001181_1292 ZM001181_1348 ZM002694_0755  ZM003358_0956 ZM003582_0482 ZM006260_0546 ZM006627_1161 ZM006954_0421 ZM006954_0886 ZM007017_0435 ZM007057_1730  ZM007565_0684 ZM007718_0376 ZM011672_0804 ZM011672_0894 ZM011877_0594 |
| 8 | ASSAY10160_00400 ASSAY10162_00160 ASSAY1024_00150 ASSAY1065_00162 ASSAY1065_00775 ASSAY1341_00252 ASSAY1399_00036  ASSAY1526_00395 ASSAY1588_00036 ASSAY1625_00118 ASSAY1660_00126 ASSAY1851_00187 ASSAY1928_00371 ASSAY1933_00182  ASSAY1977_00072 ASSAY2101_00193 ASSAY2183_00088 ASSAY2636_00222 ASSAY2814_00145 ASSAY2843_00233 ASSAY2921_00112  ASSAY3001_00298 ASSAY3010_00157 ASSAY475_00074 ASSAY498_00255 ASSAY502_00297 ASSAY558_00116 ASSAY5884_00022  ASSAY592_00056 ASSAY617_00489 ASSAY617_00569 ASSAY631_00241 ASSAY6521_00033 ASSAY6521_00611 ASSAY6799_00056 ASSAY788_00294 ASSAY869_00227 ASSAY8784_00297 ASSAY8851_00052 ASSAY8872_00327 ASSAY8941_00279 ASSAY8943_00094 ASSAY8943_00378 ASSAY8946_00112 ASSAY965_00233 AVE00086_00083 BGA00002A2_01006 BGA00005F5R5_00197 BGA00107F3R3_00229 BGA00121A1_00023 BGA00134A1_00059 BGA00275A1_00226 BGA00277A1_00151 BGA00277A1_00161 EPG00126F1R1_00730 EPG00143F1R1_00721 EPG00147F2R2_00064 EPG00147F2R2_00086 EPG00147F2R2_00260 EPG00213F3R3_00510 EPG00214F3R3_00051 EPG00214F3R3_00348 LIM00092_00296 LIM00092_00532 LIM00092_00663 LIM00092_00695 PHM15278.6 PUT-163A-29576864-1790 PUT-163A-78076151-4108 PZB01977.1 PZE-108000104 PZE-108000665 PZE-108001260 PZE-108001699 PZE-108002130 PZE-108002202 PZE-108010180 PZE-108015266 PZE-108018453 PZE-108018598 PZE-108018898 PZE-108019359 PZE-108019468 PZE-108019541 PZE-108019544 PZE-108019557 PZE-108019790 PZE-108019862 PZE-108019866 PZE-108020413 PZE-108020433 PZE-108020588 PZE-108020972 PZE-108022079 PZE-108022229 PZE-108022547 PZE-108022574 PZE-108022596 PZE-108024170 PZE-108025072 PZE-108025073 PZE-108026312 PZE-108026419 PZE-108026433 PZE-108029721 PZE-108032449 PZE-108033822 PZE-108036270 PZE-108036821 PZE-108039809 PZE-108057745 PZE-108066888 PZE-108067005 PZE-108070106 PZE-108070885 PZE-108072647 PZE-108072804 PZE-108073083 PZE-108073510 PZE-108074975 PZE-108076380 PZE-108078659 PZE-108078820 PZE-108080842 PZE-108081656 PZE-108084145 PZE-108084629 PZE-108086288 PZE-108086766 PZE-108087618 PZE-108090243 PZE-108090463 PZE-108090521 PZE-108091190 PZE-108091439 PZE-108092412 PZE-108092596 PZE-108093787 PZE-108094590 PZE-108094808 PZE-108096469 PZE-108096732 PZE-108096791 PZE-108098094 PZE-108098580 PZE-108098682 PZE-108099425 PZE-108100090 PZE-108100174 PZE-108100418 PZE-108100984 PZE-108101323 PZE-108101551 PZE-108101966 PZE-108102250 PZE-108102490 PZE-108102698 PZE-108103023 PZE-108103185 PZE-108103365 PZE-108103951 PZE-108104106 PZE-108104357 PZE-108104589 PZE-108105561 PZE-108106506 PZE-108106737 PZE-108107072 PZE-108107190 PZE-108107270 PZE-108107671 PZE-108108491 PZE-108108690 PZE-108108866 PZE-108110041 PZE-108112715 PZE-108115144 RAG00011F5R5_00359 RAG00058F1R1_00289 SYN10052 SYN10324 SYN11271 SYN11424 SYN15862 SYN18639 SYN18660 SYN19605 SYN25100 SYN31344 SYN32654 SYN35861 SYN9035 SYN9919 SYNGENTA7016 ZM000296_0502 ZM000316_0350 ZM001079_0678 ZM004075_0404 ZM004820-0602 ZM005020_0445 ZM005524_0467 ZM005524_0656 ZM005912_0452 ZM006265_1111 ZM007357_0390 ZM007733_0710 ZM008345_0433 ZM008615_0640 ZM009376_0207 ZM009801_0336 ZM010237_1012 ZM011404_0762 ZM011420_0378 ZM011878_0364 ZM012374-0236 |
| 9 | ASSAY1094_00220 ASSAY1252_00250 ASSAY1338_00040 ASSAY1397_00291 ASSAY1839_00449 ASSAY2048_00477 ASSAY2145_00113  ASSAY2339_00340 ASSAY2339_00650 ASSAY2359_00228 ASSAY2474_00068 ASSAY271_00401 ASSAY289_00638 ASSAY2961_00145 ASSAY2984_00048 ASSAY344_04453 ASSAY452_00222 ASSAY4692_00233 ASSAY4692_00320 ASSAY569_00127 ASSAY864_01047 ASSAY885_00222 ASSAY8868_00369 ASSAY980_00346 BGA00117F2R2_00083 BGA00147F2R2_00285 BGA00293F1R1_00264 EPG00138F2R2_00148 LIM00066F4R4_00028 LIM00090_00579 LIM00095_00651 LIM00143F227R227_00313 PZE-109003658 PZE-109003659 PZE-109003902 PZE-109004430 PZE-109004775 PZE-109008703 PZE-109008839 PZE-109009222 PZE-109098623 PZE-109121548 RAG00024F1R1_00146 RAG00206A1_00677 SYN19748 ZM000789_0313 ZM002285_0781 ZM003097_0921 ZM003608_0684 ZM005112_1024 ZM005314_0525 ZM005887_0752 ZM007213_0812 ZM007589_1362 ZM008236_0731 ZM009639_0328 ZM010395_0927 ZM010491_0457 ZM010851_0384 |
| 10 | ASSAY1000_00086 ASSAY1021_00410 ASSAY1957_00055 ASSAY1957_00373 ASSAY2305_00174 ASSAY2523_00222 ASSAY2523_00306  ASSAY2758_00222 ASSAY2828_00137 ASSAY2828_00185 ASSAY2861_00356 ASSAY2993_00122 ASSAY3038_00072 ASSAY3073_00150  ASSAY3169_00133 ASSAY3194_00076 ASSAY6484_00068 ASSAY6768_00407 ASSAY6768_00733 ASSAY710_00348 ASSAY718_00136 ASSAY8806_00651 ASSAY8890_00338 ASSAY950_00069 BGA00128F3R3_00291 BGA00130F1R1_00507 BGA00278F1R1_00520 BGA00278F1R1_00530 EPG00009A1_00481 EPG00009A1_00582 EPG00030F3R3_00036 EPG00119_00588 EPG00119F3R3_00021 PUT-163A-71444135-3357 PZA03570.1 PZE-110082173 PZE-110082278 PZE-110085688 PZE-110085763 PZE-110086326 PZE-110086343 PZE-110090887 PZE-110092938 PZE-110093304 PZE-110097238 PZE-110103156 PZE-110106915 SYN17783 SYN18725 SYN21905 SYN36714 SYN8162 ZM000849_0204 ZM001954_0751 ZM002884_0156 ZM005754_0332 ZM005950_0320 ZM007018_0531 ZM007148_0518 ZM007217_0521 ZM009509_0438 ZM011808_0431 |

CHR=chromosome number, SNP ID=single nucleotide polymorphic marker identity
