# Supplementary material for: Genetic diversity and population structure analyses of tropical maize inbred lines using Single Nucleotide Polymorphism markers
Source: PLoS One. 2025 Jan 24;20(1):e0315463. doi: 10.1371/journal.pone.0315463 (PMC11760008; doi:10.1371/journal.pone.0315463)
Supplement: S1 File — (ZIP) [file pone.0315463.s001.zip › Supplementary Table 2.docx]

Supplementary Table 2. SNPs used for genotyping 866 derived tropical maize inbred lines.

| CHR | SNP ID |
| --- | --- |
| 1 | PZE-101183799, SYN21642, PZE-101057118, PZE-101158245, PZE-101172177 PZE-101221583, PZE-101169548, PZE-101191970, PZE-101035737, PZE- 101001380, PZE-101091535 SYN10708, SYN35333, PZE-101195802, PUT-163A-18179375-1487, PZE-101105365 PZE-101001044, PZE-101014003, SYN21366, PZE-101067102, SYN20047, PZE-101000442, PZE-101005818, PZE-101001587, PHM3147.18, PZE-101157412, PZE-101087935, PZE-101219227, PZE-101249551, PZE-101176290, PZE-101001280, PZE-101208157, PZE-101039024, PZE-101248008, SYN21105, PZE-101167577, PZE-101195016, PZE-101095192, PZE-101019727, PZE-101017187, PZE-101024452, PZE-101206287, PZE-101003133, PZE-101134093, ZM007294-0411, PZE-101033184, ZM013367-0314, PZE-101188238, PZE101205235SYN28680, PZE-101169539, PZE-101160671, PZE-101198853, PZE-101037265, SYN32423,SYN35550 SYN14016, PUT-163A-149085801-877, PZE-101000673 PZE-101214116,PUT-163A-76287542-3906, PZE-101065728, PZE-101128157, PZE-101143143, PZE-101169204, PZE-101162393, PZE-101165146, PZE-101239020, PZE-101244459, PZE-101190874, PZE-101166671, PZE-101076890, PZE-101130082, PZE-101090347, PZE-101149674, PZE-101107138, SYN28790, PZE-101002887, PZE-101054681, PZE-101159718, PZE-101143236, PZE-101194734, PZE-101098698, PZE-101029737 SYN37775, PZE-101138790, PZE-101046118, PZE-101043507, PZE-101041588, PZE-101103210, PZE-101174535, PZE-101086075, PZA03556.2, SYN36524, SYN14771, PZE-101036991, PZE-101022796, SYN38927, PZE-101021930, PZE-101026314, PZE-101048857, PZE-101055749, SYN2735, PZE-101082812, PZE-101039207, SYN6001, PUT-163A-60355633-2771, PUT-163A-94473548-4862, PZE-101050128, PZE-101162447, PZE-101166376, PZE-101170408, PZE-101145521, PZE-101177419, PZE-101030561, PUT-163A-28982456-1669, PZE-101255156, PZE-101196520, PZE-101156159, PZA02791.9, PZE-101031077, PZE-101135107, PZE-101173330, PZE-101166237, PZE-101148240, SYN11929, PZE-101040607, PZE-101059325, PZE-101017262, PUT-163A-4647212-2116, PZE-101257120, PZE-101096356, SYN11490, SYN301, PZE-101146673, SYN6335, SYN297, SYN20364, PZE-101077708, SYN441, PZE-101055906, SYN24131, PZE-101000256, PZE-101000169, SYN23681, PZE-101063113, PZE-101123210, PZE-101215143, PZE-101069059, PZE-101177832, SYN2024, PZE-101045021, PZE-101204999, SYN9365, PZE-101059068, PZE-101041854, PZE-101039648, PZE-101035008, PUT-163A-148958166-556, PZE-101029748, PZE-101059495, PZE-101119758, PZE-101233861, PZE-101253373, PZE-101148449, PZE-101156458, PZE-101149388, SYN25458, SYN8236, SYN5537, SYN14645, PZE-101050072, PZE-101256077, SYN24213, PZE-101071564, SYN9659, PZE-101242721, PZE-101018404, PZE-101054136, PZE-101058322, PZE-101010821, SYN20196, PZE-101000349, PZE-101069159, PZE-101014266, PZE-101003026, PZE-101032246, PZE-101080712, SYN1964, PZE-101163539, SYN26731, PZE-101150128, PZE-101138952, PZE-101144969, PZE-101144427, PZE-101159305, PZE-101033489, PZE-101198848, PZE-101201013, PZE-101223466, PZE-101055223, PZE-101230194, PZE-101203040, PZE-101048892, SYN7988, PZE-101166508, PZE-101187190, PZE-101182888, SYN37548, PZE-101251928, PZE-101157898, PZE-101159556, PZE-101000740, PZE-101019557, PUT-163A-148955657-539, PZE-101018199, SYN30956, SYN2805, SYN27356, PZE-101085526, SYN37693, PZE-101096127, PZE-101093040, PZE-101204728, PZA02359.10, PZE-101033622, PZE-101256370, PZE-101011906, PZE-101038989, PUT-163A-71433079-3301, SYN13394 |
| 2 | SYN37311, PZE-102111643, PZA02175.1, PZE-102032550, PZE-102172025, PZE-102182682, PZE-102159305, SYN12020, PZE-102171071, PZE-102007609, SYN12061, PZE-102045192, PZE-102039096, PZE-102037401, PZE-102006128, PZE-102005843, PZE-102184104, PZE-102189702, PZE-102183761, PZE-102040098, PZE-110090205, SYN10369 SYN37561, PZE-102023677, SYN21949, SYN20783, PZE-110090076, PHM13440.11, PUT-163A-60342470-2456, PZE-102068646, PZA02727.1, SYN456, PZE-102184263, PZE-102175489, PZE-102182624, PZE-102006385, PZE-102185564, SYN26920, PZE-102046995, SYN26842, SYN24934, SYN4734, SYN13791, PZE-102036427, SYN4122, PZA03501.1, SYN5563, PZE-102039059, SYN18982, SYN2738, PZE-102153048, PZE-102166618, SYN24888, PZE-102186665 PZE-102178263, PZE-102017798, SYN13012, PZE-102017964, PUT-163A-28982637-1673, PZE-102151093, PZE-102179064, SYN8348, SYN5847, SYN21037, PZE-10219264, PZE-102047070, PZE-102080813, SYN28028, PZE-102179704, PZE-102067318, ZM008753-0926 PZA00620.2, PZE-102004688, SYN5848, PZE-102039991, PZE-102003235, SYN730, PZE-102145631, PZE-102056594, PZE-102000320 PHM3334.4, PZE-102027614, PZE-102016867, PZE-102047187, PZE-102006513, PZE-102035948, SYN9222, PHM3309.8, SYN25764, PZE-102168198, SYN39029, SYN20469, SYN20670, PUT-163A-76280414-3829, PZE-102170920, PZE-102122236, ABPH1.15, PUT-163A-148952002-523, PZE-102194208, PZE-102028040, PZE-102009755, PZE-102163599, SYN9524, PZE-102148457, PZE-102179936, SYN7225, PZE-102153928, SYN5809, PZE-102105276, PZE-102129725, PZE-102056425, PZE-102156525, PZE-102189664, PUT-163A-93013486-4809, PZE-10204796, PZE-102043498, SYN4131, PZE-102043269, PZE-102040312, PZE-102192129, PUT-163A-148959370-565, PZE-102118683, PUT-163A-94478236-4898, PZD00022.1, PZE-102187631, PZE-102164813, PZE-102153005, PZE-102000584, PZE-102045186, PZE-102071172, PZA00365.3, SYN6578, PZE-102166982, PZE-102158588, SYN29040, SYN38137, PUT-163A-74240952-3655, SYN21924, PZA03756.1, SYN16782, PZE-102177157, PZE-102113258, PZE-102191900, PZE-102184485, PZE-102192764, PZE-102037806, PZE-102185951, PZA02272.3, PZE-102160183, SYN8031, SYN31202, SYNGENTA11732, SYN12325, PZE-102046584, SYN7712, PZA00396.9, PZE-102170888, SYN7209, PZE-102187403, PZE-102181199, PZE-102051933, PZE-102045516, SYN11943, PZE-102194039, SYN21515, PZE-102191279, PZE-102016046, SYN216, PZE-102002451, PZE-102032700, PZE-102186688, PZE-102192809, PZE-102185011 |
| 3 | SYN30210 SYN38539, SYN5522, PUT-163A-78092706-4257, PZE-103079878, PZE-103162198, PZE-103009843, PZE-103014908, SYN39150, PZE-103116489, PZE-103144530, PZE-103080782, PZE-103073173, PZE-103031532, PZE-103024939, PZE-103127574, PZE-103127310, SYN32389, PZE-103170199, SYN26605, PZE-103138396, SYN4045, SYNGENTA16240, PZE-103024938, PZE-103133167, PZE-103103922 PZE-103148913, SYN15247, SYN6761, PZE-103100245, SYN32692, PZE-103114815, PZE-103135820, SYN35751, PZE-103119143, PZE-103007349, PZE-103011838, SYN772, PZE-103183225, PZE-103111492, SYN18034, PZE-103118666, PZE-103132991, PZE-103001968, PZE-103113298, PZE-103159840, SYN21264, PZE-103075424, PZE-10318068, PZE-103120792, PZE-103132285, PZE-103160673, PZE-103165697, PZE-103176477, PZE-103022026, ZM013362-0298, PZE-103138646, SYN20189, PZE-103021324, SYN38179, SYN33384, PZE-103073883, PZE-103072295, PZE-103165058, PZE-103078761, SYN19580, PZE-103019236, SYN31233, SYN14578, SYN31006, PZE-103107305, SYN34621, SYN33799, PUT-163A-14245173-339, PZE-103116584, SYN33866, SYN5650, SYN32818, PZE-103143600, PZE-103175946, PZE-103173858, PZE-103138454, PUT-163A-18172180-1378, PZE-103157756, SYN1545, SYN11886, SYN28626, PZE-103119311, SYN15340, SYN35425, PZE-103183915, SYN34629, SYN34245, PZE-103029035, PZE-103089806, SYN7429, PZE-103181867, PZE-103091618, PZE-103110810, PZE-103125173, PZE-103124207, PZE-104132531, PZE-103183701, PZE-103102419, PZE-103029988, PZE-103149272, PZE-103081138, SYN14789, PUT-163A-71331763-3192, PZE-103129508, PUT-163A-71423695-3223,, PZE-103175094 PZE-103088324, PZE-103108142, PZE-103005351, PZE-103015842, PZE-103166903, PZE-103165581, SYNGENTA3276, PZE-103094153, PZA00316.9, PZE-103159288, PZE-103162582, PZE-103148259, PZE-103171593, PZE-103034362, PZE-103136532, PZE-103037509, PZE-103179063, PZE-103123844, PZE-103154125, PZB01109.2, PZE-103125005, PZE-103133286, PZE-103184089, PZE-103185158, PZE-103102170, PZE-103140146, SYN36904, PZE-103182817, PZE-103124740, SYN23204, PZE-103151247, PZE-103023737, PZE-103100557, PZE-103013845, PZE-103013339, PZE-103008604, PZE-103000497, PZE-103021311, PZE-103155455, PZE-103177259, PZE-103166830, PZB02510.5, PZE-103106233, PZE-103186797, PZE-103186551, PZE-103009187, PZE-103182570, SYN26962, PZE-103019131, PZE-103141413, SYN25915, PZE-103167850, PZE-103129454, PZE-103125818, PZE-103186172, PZE-103075693, SYN1528, PZE-103178956, PZE-103162731, PZE-103179078, PZE-103150048, PZE-103115210, PZE-103132593, PZE-103167510, SYN36244, PZE-103006828, PZE-103001192, SYN6775, PZE-103136668, PZE-103014951, PZE-103074862, SYN10949, PZE-103102712, PZE-103010658, PZE-103030646, PZE-103126437, PZE-103016707 |
| 4 | PZE-104151980, PUT-163A-110206849-64, PZE-104122684, PZE-104007604, SYN16455, PZE-104021862, PZE-104073544, PZE-104015374, PZE-104019190, PZE-104112675, PZE-104103734, PZE-104146173, PZE-104096416, PZE-104092819, PZE-104153707, PZE-104033608, PZE-104141018, PZE-104010129, PZE-104022829, PZE-104110016, PZE-100002530, ZM005465-1585, PZE-104033817, SYN24904, PZE-104022542, PZE-104128792, PZE-104109023, PZE-104148799, SYN8509, PZE-104048303, PZE-104005331, PZE-104073340, PZE-104099276, PZE-104092741, PZE-104157368, PZE-104073719, PZE-104017018, PZE-104097152, PZE-104102755, PZE-104008143, SYN7340, PZE-104155284, PUT-163A-78115415-4344, PZE-104103558, PZE-104016598, PZE-104123124, PZE-104126595, PZE-104135135, PZE-104021091, PZE-104087964, PZE-104106790, PZE-104109208, PZE-104001404, PZE-104005031, PUT-163A-6021631-2317, PZE-104000045, PZE-104113793, PZE-104078833, PZE-104101474, PZE-104107792, PZE-104106634, PZE-104154748, PZE-104092771 SYN24015, PZE-104012818, PZE-104012490, PZE-104024520, PZE-104094616, PUT-163A-148946720-494, SYN28884, PZE-104011650, PZE-104126415, PZE-104152590, PZE-104151091, SYN1980, SYNGENTA17524, PZE-104077825, PZE-104125636, PZE-104097453, PZE-104105055, PZE-104121843 PZE, 104017065, SYN2344, PUT-163A-76016170-3772, PZE-104114501, ZM008539-0453, PZE-104137088,, PZE-104110543 PZE-104156376, PZE-104101343 SYN4601, PZE-104089679, PZE-104076811, PZE-100002529, PZE-104005531, PZE-104152999, PZE-104157783, PZE-104131042, PZE-104144717, PZE-104021108, PZA00086.8, PZE-104077581, PZE-104139498, PZE-104139266, PZA02385.6, PZE-104151042, PZE-104128955, ZM013591-0465, SYN17723, PZE-104003896, PZE-104006040, PZE-104106986, PZE-104100716, PUT-163A-78113253-4315, PZE-104118540, PZE-104023748, PZA02239.11, PZE-104154015, PZE-104086911, PZE-104144719, PZE-104099687, ZM013700-0405, PZE-104067991, PZE-104090796, PZE-104009499, PZE-104076568, SYN8790 SYN23006, PZE-104079748, PZE-104014765, PZE-104009398, PZE-104013781, PZE-104097989, PZE-104001592, PUT-163A-60354034-2733, SYN29285, SYN18894, PZE-104114732, PZE-104003437, PZE-104022139, PZE-104001411, PZE-104043634, SYN2451, PZE-104103423, SYN29997, SYNGENTA13943, SYN28711, PZE-104009983 SYN34551, PZE-104010497, SYN8706 |
| 5 | PZE-105169286, PZE-105163488, PZE-105152260, PUT-163A-91050968-4727, SYN27136, SYN31305, PZE-105162073, PZE-105037748, SYN5363, PZE-105002105, PZE-105039646, PZE-105157658, PZE-105022516, PUT-163A-16925044-1085, PZE-105023037, PUT-163A-16925922-1125, PZE-105180266, SYN9873, PZE-105123694, PZE-105115795, SYN11021, SYN36770, PZE-105110864, SYN4379, PZE-105083729, SYN9389, SYN29224, PZE-105016296, SYN29192, PZE-105026024, PZE-105143481, SYN38466, SYN28081, SYN1045, PZE-105014188, PZE-105026200, PZE-105019032, PUT-163A-60395273-2925, SYN12357, SYN22488, PZE-105108884, PZE-105000100, SYN38866, PZE-105009142, PZE-105069286, PZE-105003814, SYN1406, PZE-105017654, PZE-105022751, SYN2910, PZE-105019465, SYN21612, PZE-105004767, ZM010996-0545, SYN30171, PZA00987.1, SYN35170, PZE-105019472, SYN903, PZE-105014190, SYN25255, PZE-105012348, PUT-163A-28985129-1703, PZE-105016060, PZE-105161112, SYN9166, PZA00448.6, PZE-105018859, PZE-105165764, SYN9171, PZE-105031680, PZE-105177822, PZE-105166721, PZE-105072012, SYNGENTA16696, ZM013424-0538, PZE-105065040, PZE-105022450, PZE-105169468, PZE-105123635, PZE-105006205, PZE-105011866, PZE-105156713, PZE-105182093, SYN20663, PZE-105107160, PZE-105098019, PZE-105019536, PZE-105040420, PZE-105086310, PZE-105133858, PZA00985.1, PZA-002390001, PZE-105025224, SYN35950, PZE-105024535, SYN9877, PUT-163A-101389210-16, PZE-105074389, PZE-105166980, PZE-105028038, PZE-105044284, PZE-105163590, PZE-105160758, PZE-105035278, PZE-105066407, PZE-105048907, PZE-105182641, PZE-105056273, PZE-105158473, PUT-163A-76285192-3869, ZM013489-0395, SYN32082, PZE-105046069, PZE-105156970, PZE-105160408, PZE-105158980, PZE-105109854, PUT-163A-89763149-4708, PZE-105060590, PUT-163A-148962814-587, PZE-105130181, PZE-105128589, PZE-105114709, SYN22185, PZE-105126583, PZE-105150220, PZE-105114994, PZE-105147471, PZE-105122422, PZE-105122072, PZE-105145814, PZE-105015838, PZE-105180267, PZE-105165114, SYN28053, SYN27814, PZE-105156596, PZE-105127434, PZE-105113974, PZE-105134993 |
| 6 | PZE-106063281, PZE-106021194, PZE-106072217, SYN3491, PZE-106082237, PZE-106092483, PZE-106054425, SYN21816, PZE-106001089, PZE-106087191, PZE-106088503, PZE-106084808, PZE-106060834, PZE-106111799, PZE-106061456, PZE-106038186, PUT-163A-18163247-1246, PZE-106057058, PZE-106050075, PZE-106080884, PZE-106105801, PZE-106000507, PZE-106001126, PZE-106129840, PZE-106062474, PZE-106131218, PZE-106056649, SYN4151, PZE-106130082, PZE-106050102, PZE-106090096, SYN24412, PZE-106008406, PZE-106044976, PZE-106084109, PZE-106092530, PZE-106128641, SYN35815, SYN7863, SYN11825, PZE-106050790, PZE-106093879, SYNGENTA11851, SYN10787, PZE-106123512, PUT-163A-26557108-1602, SYN10494, PZE-106064449, PZE-106068309, PZE-106058198, PZE-106065440, PZE-106048775, PZE-106075587, PUT-163A-71769572-3542, PZE-106065688, PZE-106097584, SYN23631, PZE-106126667, SYN26189, SYN10686, ZM007490-0484, SYN26240, SYN17152, PUT-163A-13237300-197, PZE-106050053, SYN8250, SYN11442, SYN9304, PZE-106051785, SYNGENTA16367, PZE-106054274, PZE-106020716, PUT-163A-18169220-1336, PZE-106003908, SYN10489, PZE-106086978, PZE-106128640, SYN24540, PZE-106007551, PZE-10611535, PZE-106008070, PUT-163A-31909945-2001, PZE-106114611, PZE-106043252 PZE-106070134, PUT-163A-78119421-4381, PZE-106016519, PHM3466.69, PZE-106102127, PZE-106059535, PZE-106001449, PZE-106001672, SYN4177, PZE-106068510, PZE-106055176, PZE-106086811, SYNGENTA10895, PZE-106095314, PZE-106088879, SYN34382, PZE-106115402, PZE-106069853, PZE-106099144, PZE-106058481, SYN20087, PZE-106077744, PZE-106100715, PZE-106004351, PZE-106000131, PZE-106020572, SYN13503, SYN35814, PZE-106108243, PZE-106128992, PZE-106119113, PZE-106115028, PZE-106030011 |
| 7 | PZE-107105917, PZE-107077547, PZE-107073339, PZE-107010573, PZE-107004786, PZE-107072999, SYN12247, PZE-107010433,SYN17085, PZE-107013546, PZE-107084721, ZM011374-0383, PZE-107043379, PZE-107129950, PZE-107021172, PUT-163A-60347260-2574, SYN29618, SYN13029, PZE-107004845, SYN18954, SYNGENTA5472, PZE-107017571, PZE-107013187, SYN18602, PZE-107100880, SYN17053, PZE-107000362, PZE-107098286, PZE-107093186, PZE-107025223, PZE-107113353, SYN12221, PZE-107128144, SYN34204, SYN36195, SYNGENTA6440, PZE-107005831, SYN20419, ZM012706-0416, PZE-107068214, PZE-107015809, SYN14546, PUT-163A-4573196-2068, PZE-107100610, PZE-107063357, PZE-107012088, PZE-107046723, ZM008047-0510, PZE-107110616, SYN34652, PZE-107117400, SYN37148, PZE-107127637, PZE-107121188, PZE-107102004, PZE-107113201, SYN3390, PZE-107081140, PZE-107015950, SYN3230 PZB01083.1, SYN23691, SYN10741, PZE-107137037, SYN29953, SYN23679, PZE-107060543, SYN32824, PZE-107072025, PZE-107074405, PZE-107015811, PZE-107113723, PZE-107069959, PZE-107108200, PZE-107075781, PZE-107065748, PZE-107000059, PZE-107104221, PZE-107096067, PZE-107081355, PZE-107106472, PZE-107009208, PZE-107011738, PZE-107010578, PZE-107042998, SYN13265, PZE-107130514, PZE-107051302, PZE-107065723, PZE-107091643, PZE-107085004, PZE-107136092, PZE-107130565, PZE-107110721, PZE-107128145, PZE-107084474, PZE-107082389, PZE-107123019, PZE-107128845, PZE-107078697, PZE-107010082, PZE-107135769, PZE-107078834, PZE-107120886, PZE-107113337, PZE-107123571, PZE-107059010, PZE-107103294, SYN6902, PZE-107023596, SYNGENTA5469, PZE-107108975, PZE-107021428, SYN2766, SYN6948, SYN5002, PZE-107054011, PZE-107066917, PZE-107132730, PZE-107134212, SYN18482, PZE-107128646, SYN17015, PZE-107137944, SYN15673, PZE-107138216, SYN37961, SYN24605, ZM006162-0457, PZE-107016512, PZE-107002330, PZE-107019079, PZE-107017355, PZE-107050584, PZE-107015084 |
| 8 | PZE-108106737 PUT-163A-74233701-3600 PZE-108115144 PZE-108018114 PZE-108068459 PUT-163A-71425880-3235 PZE-108003848 PZE-108112715 PZE-108005438  PZE-108021716, PZE-108135749, SYN29240, SYN11271, PZE-108115325, PZE-108059057, PZE-108059579, PZE-108135190, PZE-108127168, PZE-108118864, PZE-108051264, SYN18342, SYN36002, SYN25100, PZE-108057885, PZA00001.8, PZE-108106506, PZE-108003557, SYN27235, PZE-108022229, PZE-108086766, PZE-108103365, PZE-108055029, PUT-163A-148961306-579, PZE-108068669, PZE-108054764, SYN15862, PZA-000516003, PZE-108067005, PZE-108000028, PZE-108004863, PZE-108099332, PZE-108097250, PZE-108009465, PZE-108005561, PZE-108081297, PZE-108135669, PZE-108060935, PZA00058.1, PZE-108133641, PZE-108108866, PZE-108097569, PZE-108000108, PZE-108000394, PZE-108110343, PZE-108045430, PZE-108077809, SYN23220, PZE-108133176, PZE-108056050, PZE-108042918, PZE-108062259, PZE-108016169, PZE-108071899, PZE-108104835, PZE-108005632, SYN26310, PZE-108007793, PZE-108108491, PZE-108118870, PUT-163A-13557719-256, PZE-108101678, PZE-108014370, PZE-108009299, PZE-108004350, PUT-163A-4647150-2110, PUT-163A-76010623-3722, PZE-108004841, SYN23659 PZE-108017647, PZE-108008825, PZE-108025068, PZE-108100840, PZE-108073336, PUT-163A-78093215-4263, PZE-108024244, SYN34541, ZM013954-0344, PZE-108058793, PZE-108046584, PZE-108065360, PZE-108078659, SYN7584, RF4, PZE-108094138, PZE-108120405, SYN20700, PZE-108057745, PZE-10805641, PZA03592.3, SYN9027, SYN20808 PZE-108081128, SYN19146, PZE-108113165, PZE-108105216, PZE-108053798, PUT-163A-18172270-1381, PZE-108018134, PZE-108062040, PZE-108116469, PZE-108054980, PZE-108117488, PZE-108110152, PZE-108071872, SYN2782, PZE-108018897, PZE-108004315, PZE-108017470, PZE-108011210, PZE-108020640, PZE-108014231, PZE-10803175, PZE-108036758, PZE-108052678, SYN13074, PZE-108133100, PUT-163A-18173735-1405, PZE-108007891, PZE-108016243, PZE-108070762, PZE-108043299, PZE-108061338, PZE-108058442, PZE-108012648, PZE-108018598, SYN26248, SYN8904, PZE-108016244, PZE-108112124, PZE-108070106, PZE-108006522 |
| 9 | SYN7122, PZE-109121844 SYN26333, PZE-109085594, PZE-109008718, PZE-100002298, PZE-109094881, PZE-109016739, PZE-109104845, PUT-163A-60357079-2822, PZE-109035289, PZE-109051898, PZE-109121548, PZE-109081862, PZE-109012469, SYN35320, PZE-109052349, PZE-109009763, SYN12315, PZE-109001491, PZE-109109509, PZE-109098623, PZE-109003441, PZE-109056189, PZE-109054205, PZE-109098305, SYN27145, PZE-109120935, PZE-109019092, SYN4009, PZE-109075835, PZE-109110455, PZE-109004241, SYN3709, PZE-109011432, SYN5447, PZE-109073279, PUT-163A-148928873-366, PZE-109113692, PZE-109101217, PZE-109001692, PZE-109063960, PZE-109011840, PUT-163A-94480077-4901, PZE-109020723, PZE-109015397, PZE-109016499, PZE-109073072, PZE-109022064, SYN5116, PZE-109059086, SYN7324, PUT-163A-78075531-4099, PZE-109092637, PZE-109009258, SYN13950, PZE-109001250, PZE-109061149, PZE-109117925, PZE-109107248, PZE-109115639, PZE-109109278, PZE-109003902, PUT-163A-110541282-116, SYN5443, PZE-109090152, PZE-109093918, PZE-109027147, ZM009290-0678, PZE-109109991, SYN34181, SYN22241, SYN5732, PUT-163A-50330000-2208, PZE0003716573, PZE-109109841, PZE-109121611, PZE-109121159, PZE-109083328, PZE-109066188, PZE-109090188, PZE0003747944, PZE-109106186, SYN6090, PZE-109107501, SYN7150, SYN13447, PZE-109085093, PZE-109069453, SYN6666, PZE-109034802, PZE-109121439, PZB01963.6, PZE-109016177, PZE-109054389, PZE-109002663, PZE-109022525, PZE-109015063, PZE-109021565, PZE-109000128, PZE-109010665, PZE-109119987, PZE-109080508, PZE-109106291, PZE-109085791, PZE-109106589, PZE-109009221, SYN34947, PZE-109082542, PZE-109068629, PZE-109018303, PZE-109027642, PZE-109013123, SYN6935, SYN30121, SYN9773, PZE-109007822, PZE-109099670, PZE-109116158, PZE-109009987, PZE-109103538, PZE-109113250, PZE-109114000, SYN27711, PZE-109101246, PZE-109101698, PZE-109098108, PZE-109015136, PZE-109006355, PZE-109066006 |
| 10 | PZE-110102769, PZE-110084114, PZE-110079903, PZE-110109364, SYN17753, PZE-110097238, PZE-110104616, PZE-110014434 PZE-110013181, PZE-110083604, PZE-110058551, PZE-110070387, PZE-110069014, PZE-110034877, PZE-110103156, SYN25426, SYN8538, PZE-110090412, PZE-110092938, PZE-110043016, SYN17213, PZE-110009498, SYN10789, PZE-110000614, PZE-110111368, PZE-110110463, SYN9690, PZE-110009173, PZE-110047509, SYN39326, PZE-110076759, PZE-110008044, PZE-110097954, PZE-110085234, SYN448, PZE-110079535, PZE-110039531, PZE-110099063, PZE-110106767, PZE-110099681, PZE-110026433, PUT-163A-71444135-3357, ZM011839-0709, PZE-110052967, PZE-110050825, PZE-110010070, PUT-163A-29544531-1779, SYN18729, PZE-110066518, PZE-110001270, PZE-110069146, SYN18725, PZE-110061194, PZE-110088808, PZE-110014712, ZM012507-0197, PZE-110007326, PZE-110020077, PZE-110042009, PZE-110101412, PZB02237.1, PZE-110093416, PZE-110009748, PZE-110085769, PZE-110076077, PZE-110044605, SYN22564, PZE-110097661, SYN37374, PZE-110008882, PZE-110100195, PZE-110100685, SYN21905, SYN17783, PZE-110026520, PZE-102187687 |

CHR=chromosome number, SNP ID=single nucleotide polymorphic marker identity
