# Supplementary material for: Genetic diversity and population structure analyses of tropical maize inbred lines using Single Nucleotide Polymorphism markers
Source: PLoS One. 2025 Jan 24;20(1):e0315463. doi: 10.1371/journal.pone.0315463 (PMC11760008; doi:10.1371/journal.pone.0315463)
Supplement: S1 File — (ZIP) [file pone.0315463.s001.zip › Supplementary Table 3.docx]

Supplementary Table 3. Codes for the 182 founder parental inbred lines of maize.

| **Line Code** | **Code description** |
| --- | --- |
| 1 to 10 | RGS-PL65, RGS-PL08, RGS-PL09, RGS-PL68,RGS-PL63, RGS-PL62, RGS-PL38, RGS-PL53, 16ARG16812, RGS-PL20 |
| 11 to 20 | RGS-PL10, RGS-PL58, RGS-PL06, 15ARG119, 15ARG142, 15ARG143, |
|  | 15ARG148, 15ARG149, 15ARG151, 15ARG175 |
| 21 to 30 | RGS-PL28,16ARG16786, 16ARG16788, 16ARG16791, 16ARG16792, |
|  | 16ARG16804,16ARG16816, RGS-PL23, RGS-PL48,RGS-PL52 |
| 31 to 40 | 15ARG104, 15ARG176, RGS-PL30, 16ARG16798, 16ARG16815, |
|  | RGS-PL54, RGS-PL19, RGS-PL17, 15ARG112, 15ARG127 |
| 41 to 50 | 15ARG129, 15ARG152, RGS-PL60, RGS-PL13 , 16ARG16782, 16ARG16814, RGS-PL07, RGS-PL25, 15ARG111, 15ARG114 |
| 51 to 60 | 15ARG132, 15ARG140, 15ARG161, RGS-PL66, RGS-PL11, RGS-PL71, |
|  | RGS-PL36, RGS-PL01, 16ARG16784, 16ARG16795 |
| 61 to 70 | RGS-PL44, 15ARG110, 15ARG123, 15ARG164, RGS-PL31, RGS-PL29, |
|  | RGS-PL43, 16ARG16794, RGS-PL47, RGS-PL69 |
| 71 to 80 | 15ARG117, 15ARG128, 15ARG158, 15ARG160, 15ARG173, 16ARG180, |
|  | 16ARG16790, 16ARG16802, 16ARG16806, 16ARG16811 |
| 81 to 90 | RGS-PL50, RGS-PL03, RGS-PL59, 15ARG131, 15ARG137, 15ARG147, |
|  | 15ARG154, 15ARG155, 15ARG159, 15ARG165 |
| 91 to 100 | 15ARG167, 15ARG174, RGS-PL27, RGS-PL33, 16ARG178, |
|  | 16ARG16803, 16ARG16807, RGS-PL18, RGS-PL24, 15ARG106 |
| 101 to 110 | 15ARG107, 15ARG116, 15ARG125, 15ARG126, 15ARG130, 15ARG144, |
|  | 15ARG153, 15ARG171, RGS-PL05, RGS-PL45 |
| 111 to 120 | 16ARG16781, 15ARG121, 15ARG124, 15ARG157, 15ARG168, |
|  | RGS-PL64, 16ARG16789, 16ARG16796, 16ARG16797, 16ARG16801 |
| 121 to 130 | 16ARG16805, RGS-PL56, RGS-PL55, RGS-PL15, RGS-PL21, |
|  | RGS-PL02, 15ARG145, 15ARG163, 15ARG172, 16ARG181 |
| 131 to 140 | 16ARG16800, 15ARG109, 15ARG120, 15ARG122, 15ARG136, |
|  | 15ARG138, 15ARG166, 15ARG177, RGS-PL14, 16ARG16785 |
| 141 to 150 | 16ARG16809, 16ARG16817, RGS-PL26, RGS-PL16, RGS-PL40 |
|  | 15ARG113, 15ARG115, 15ARG150, 15ARG156, RGS-PL57 |
| 151 to 160 | RGS-PL61, RGS-PL49, RGS-PL37, 16ARG16793, RGS-PL12, |
|  | RGS-PL22, RGS-PL04, RGS-PL39, 15ARG105, 15ARG133 |
| 161 to 170 | 15ARG135, 15ARG139, 15ARG169, RGS-PL70, RGS-PL42, |
|  | 16ARG16799, 16ARG16808, 15ARG103, 15ARG146, 15ARG170 |
| 171 to 182 | RGS-PL67, 16ARG179, 16ARG16787, 16ARG16810, 16ARG16813, |
|  | RGS-PL41, RGS-PL46, 15ARG108, 15ARG118, 15ARG134, 15ARG141, |
|  | 15ARG162 |
