# Supplementary material for: Genetic diversity and population structure analyses of tropical maize inbred lines using Single Nucleotide Polymorphism markers
Source: PLoS One. 2025 Jan 24;20(1):e0315463. doi: 10.1371/journal.pone.0315463 (PMC11760008; doi:10.1371/journal.pone.0315463)
Supplement: S1 File — (ZIP) [file pone.0315463.s001.zip › Supplementary Table 4.docx]

Supplementary Table 4. Distribution of the 182 founder parental lines of maize into three subpopulations.

| **Cluster** | **Line** |
| --- | --- |
| 1 | 15ARG164 RGS-PL19 RGS-PL12 15ARG120 15ARG170 15ARG139 15ARG151 15ARG105 |
|  | 15ARG103 15ARG135 15ARG111 15ARG138 15ARG104 RGS-PL50 15ARG131 15ARG159 |
|  | RGS-PL39 15ARG122 15ARG142 15ARG140 15ARG158 15ARG173 15ARG145 RGS-PL01 |
|  | 15ARG167 15ARG161 RGS-PL47 15ARG163 15ARG143 15ARG148 15ARG147 15ARG112 |
|  | 15ARG127 15ARG129 15ARG121 15ARG110 15ARG123 15ARG117 |
| 2 | RGS-PL65 RGS-PL08 RGS-PL09 RGS-PL68 RGS-PL63 RGS-PL62 RGS-PL38 RGS-PL53 |
|  | RGS-PL20 RGS-PL10 RGS-PL58 RGS-PL06 RGS-PL23 RGS-PL48 RGS-PL52 15ARG176 |
|  | RGS-PL30 RGS-PL54 RGS-PL17 RGS-PL60 RGS-PL13 RGS-PL07 RGS-PL25 15ARG132 |
|  | RGS-PL66 RGS-PL11 RGS-PL71 RGS-PL36 RGS-PL44 RGS-PL31 RGS-PL29 RGS-PL43 |
|  | RGS-PL69 15ARG128 15ARG160 RGS-PL03 15ARG137 15ARG149 15ARG154 15ARG155 |
|  | 15ARG165 15ARG174 RGS-PL27 RGS-PL33 16ARG178 RGS-PL18 RGS-PL24 15ARG106 |
|  | 15ARG107 15ARG116 15ARG125 15ARG126 15ARG130 15ARG144 15ARG153 15ARG171 |
|  | RGS-PL05 RGS-PL45 15ARG152 15ARG124 15ARG157 15ARG168 RGS-PL64 RGS-PL56 |
|  | RGS-PL55 RGS-PL15 RGS-PL21 RGS-PL02 15ARG172 15ARG109 15ARG136 15ARG166 |
|  | 15ARG177 RGS-PL14 RGS-PL26 RGS-PL16 RGS-PL40 15ARG113 15ARG115 15ARG150 |
|  | 15ARG156 RGS-PL57 RGS-PL61 RGS-PL49 RGS-PL37 RGS-PL22 RGS-PL04 15ARG133 |
|  | 15ARG169 RGS-PL70 RGS-PL42 15ARG146 RGS-PL67 RGS-PL41 RGS-PL46 15ARG108 |
|  | 15ARG118 15ARG134 15ARG141 15ARG162 |
| 3 | RGS-PL28 16ARG16787 15ARG114 16ARG16817 15ARG119 16ARG16792 15ARG175 16ARG16815 |
|  | 16ARG16814 16ARG16811 RGS-PL59 16ARG16804 16ARG16782 16ARG16786 16ARG16798 |
|  | 16ARG16784 16ARG16795 16ARG16794 16ARG16781 16ARG16796 16ARG16797 16ARG16801 |
|  | 16ARG16803 16ARG16807 16ARG16785 16ARG16809 16ARG16812 16ARG16791 16ARG16800 |
|  | 16ARG16793 16ARG16799 16ARG16808 16ARG16810 16ARG16813 16ARG16806 16ARG16805 |
|  | 16ARG179 16ARG16789 16ARG16790 16ARG16788 16ARG16816 16ARG16802 16ARG181 |
|  | 16ARG180 |
