# Supplementary material for: Genetic diversity and population structure analyses of tropical maize inbred lines using Single Nucleotide Polymorphism markers
Source: PLoS One. 2025 Jan 24;20(1):e0315463. doi: 10.1371/journal.pone.0315463 (PMC11760008; doi:10.1371/journal.pone.0315463)
Supplement: S1 File — (ZIP) [file pone.0315463.s001.zip › Supplementary Table 5.docx]

Supplementary Table 5. Two clusters of the 866 derived maize inbred lines based on SNP analysis.

| **Cluster** | **Genotypes** |
| --- | --- |
| 1 | G15NL357 G15NL288 G16NL42 G16NL71 G16NL83 G16NL93 G16NL94 G16NL95 G16NL130 G16NL153 |
|  | G17NL182 G17NL184 G17NL225 G18NL262 G18NL264 G15NL348 G15NL350 G15NL351 G15NL360 |
|  | G15NL299 G18NL206 G16NL89 G16NL91 G16NL97 G16NL105 G16NL106 G16NL114 G16NL132 |
|  | G16NL156 G17NL166 G17NL174 G17NL175 G17NL191 G18NL276 G15NL04 G15NL32 G15NL36 |
|  | G15NL346 G15NL291 G15NL295 G16NL72 G16NL99 G16NL109 G16NL119 G16NL126 G17NL157 |
|  | G17NL196 G17NL213 G17NL218 G17NL222 G17NL228 G17NL237 G18NL246 G15NL27 G15NL362 |
|  | G15NL313 G15NL361 G15NL283 G16NL48 G16NL59 G16NL81 G16NL125 G16NL150 G17NL159 |
|  | G17NL192 G17NL194 G17NL207 G17NL214 G17NL505 G17NL234 G18NL274 G15NL16 G15NL308 |
|  | G15NL281 G15NL297 G15NL298 G16NL38 G16NL46 G16NL60 G16NL61 G16NL684 G16NL73 G16NL100 |
|  | G16NL123 G16NL124 G16NL149 G17NL202 G17NL221 G17NL231 G17NL233 G18NL253 G18NL263 |
|  | G15NL367 G15NL368 G15NL310 G15NL330 G15NL331 G15NL344 G15NL354 G15NL294 G16NL62 |
|  | G16NL65 G16NL67 G16NL78 G16NL85 G16NL86 G15NL12 G15NL14 G15NL31 G15NL306 G15NL312 |
|  | G15NL320 G16NL43 G16NL54 G16NL57 G16NL63 G16NL107 G17NL163 G17NL170 G17NL178 |
|  | G17NL179 G17NL200 G17NL193 G17NL209 G17NL210 G17NL212 G17NL220 G16NL136 G16NL142 |
|  | G18NL266 G15NL05 G15NL26 G15NL364 G15NL370 G15NL309 G15NL334 G15NL336 G15NL342 |
|  | G16NL50 G16NL66 G16NL75 G16NL96 G16NL98 G16NL133 G17NL187 G17NL189 G17NL198 G17NL199 |
|  | G17NL215 G18NL248 G18NL249 G18NL257 G18NL261 G18NL270 G18NL280 G15NL03 G15NL09 |
|  | G15NL327 G15NL332 G15NL341 G15NL304 G15NL356 G17NL223 G17NL188 G17NL176 G17NL197 |
|  | G17NL219 G17NL236 G17NL242 G18NL243 G15NL07 G15NL19 G15NL23 G15NL30 G15NL326 |
|  | G15NL329 G15NL339 G15NL347 G15NL353 G16NL37 G16NL39 G16NL47 G16NL49 G16NL104 |
|  | G16NL134 G17NL160 G17NL185 G17NL186 G17NL195 G17NL201 G17NL232 G18NL254 G18NL258 |
|  | G18NL265 G17NL235 G18NL244 G18NL245 G18NL251 G18NL278 G18NL676 G15NL01 G15NL15 |
|  | G15NL318 G15NL323 G16NL40 G16NL56 G16NL64 G16NL88 G16NL122 G16NL131 G16NL151 |
|  | G16NL148 G17NL167 G17NL168 G17NL203 G17NL208 G17NL211 G17NL226 G17NL241 G18NL252 |
|  | G18NL267 G18NL277 G15NL10 G15NL314 G15NL301 G15NL340 G15NL285 G16NL41 G16NL90 |
|  | G16NL108 G16NL112 G16NL138 G16NL143 G17NL217 G17NL229 G18NL259 G15NL08 G15NL18 |
|  | G15NL22 G15NL319 G15NL302 G15NL355 G15NL305 G15NL289 G15NL296 G16NL55 G16NL68 |
|  | G16NL70 G16NL101 G16NL102 G16NL115 G16NL120 G16NL155 G17NL164 G17NL173 G18NL273 |
|  | G18NL275 G15NL365 G15NL303 G15NL338 G15NL352 G15NL286 G16NL44 G16NL127 G16NL137 |
|  | G16NL141 G16NL144 G16NL152 G17NL158 G17NL177 G17NL190 G18NL260 G18NL269 G15NL06 |
|  | G15NL25 G15NL33 G15NL328 G15NL333 G15NL292 G15NL293 G16NL58 G16NL79 G16NL118 |
|  | G16NL121 G16NL135 G16NL117 G16NL129 G17NL162 G17NL216 G18NL250 G18NL271 G18NL279 |
|  | G15NL02 G15NL20 G15NL35 G15NL363 G15NL316 G15NL324 G15NL337 G15NL349 G15NL284 |
|  | G16NL45 G16NL87 G16NL103 G16NL145 G17NL161 G17NL172 G17NL183 G17NL205 G17NL238 |
|  | G17NL239 G15NL17 G15NL34 G15NL369 G15NL307 G15NL317 G15NL300 G15NL321 G15NL325 |
|  | G15NL359 G15NL287 G16NL74 G16NL80 G16NL128 G16NL147 G17NL165 G17NL204 G16NL113 |
| 2 | G16NL690 G16NL681 G16NL765 G16NL773 G16NL807 G16NL870 G16NL871 G16NL886 G16NL887 |
|  | G16NL920 G17NL438 G17NL637 G17NL663 G17NL456 G17NL466 G17NL471 G17NL477 G17NL500 |
|  | G17NL537 G17NL550 G17NL556 G17NL560 G17NL584 G17NL586 G17NL597 G18NL668 G18NL671 |
|  | G16NL713 G16NL778 G16NL785 G16NL787 G16NL791 G16NL811 G16NL820 G16NL821 G16NL826 |
|  | G16NL828 G16NL849 G16NL856 G16NL857 G16NL865 G16NL889 G17NL377 G17NL415 G17NL417 |
|  | G17NL425 G17NL653 G17NL488 G17NL595 G18NL665 G18NL669 G18NL674 G16NL686 G16NL714 |
|  | G16NL719 G16NL721 G16NL722 G16NL756 G16NL789 G16NL815 G16NL914 G16NL824 G16NL881 |
|  | G16NL901 G16NL918 G17NL625 G17NL395 G17NL412 G17NL436 G17NL631 G17NL644 G17NL655 |
|  | G17NL442 G17NL446 G17NL497 G17NL509 G17NL513 G17NL529 G18NL673 G16NL678 G16NL697 |
|  | G16NL701 G16NL704 G16NL706 G16NL729 G16NL739 G16NL744 G16NL784 G16NL790 G16NL819 |
|  | G16NL825 G16NL840 G16NL845 G16NL846 G16NL902 G16NL903 G17NL374 G17NL388 G17NL399 |
|  | G17NL408 G17NL636 G17NL453 G17NL533 G17NL540 G17NL559 G17NL567 G17NL602 G17NL623 |
|  | G18NL670 G16NL698 G16NL758 G16NL796 G16NL799 G16NL812 G16NL913 G16NL834 G16NL839 |
|  | G16NL859 G16NL895 G17NL373 G17NL378 G17NL381 G17NL414 G17NL418 G17NL420 G17NL439 |
|  | G17NL639 G17NL646 G17NL648 G17NL448 G17NL462 G17NL495 G17NL502 G17NL510 G17NL522 |
|  | G17NL525 G17NL563 G17NL568 G17NL578 G17NL591 G16NL694 G16NL700 G16NL702 G16NL708 |
|  | G16NL709 G16NL725 G16NL727 G16NL742 G16NL750 G16NL793 G16NL800 G16NL801 G16NL802 |
|  | G16NL843 G16NL858 G16NL861 G16NL862 G16NL872 G16NL908 G16NL916 G17NL375 G17NL396 |
|  | G17NL422 G17NL435 G17NL645 G17NL450 G17NL451 G17NL460 G17NL464 G17NL479 G17NL511 |
|  | G17NL515 G17NL574 G17NL575 G17NL581 G17NL587 G17NL609 G16NL715 G16NL716 G16NL723 |
|  | G16NL734 G16NL735 G16NL738 G16NL745 G16NL751 G16NL759 G16NL766 G16NL776 G16NL780 |
|  | G16NL797 G16NL806 G16NL836 G16NL853 G16NL884 G16NL888 G16NL891 G16NL893 G16NL898 |
|  | G17NL384 G17NL391 G17NL392 G17NL401 G17NL402 G17NL428 G17NL437 G17NL630 G17NL651 |
|  | G17NL443 G17NL445 G17NL457 G17NL467 G17NL489 G17NL503 G17NL514 G17NL527 G17NL561 |
|  | G17NL565 G17NL580 G17NL588 G17NL620 G17NL624 G18NL672 G17NL657 G17NL664 G17NL496 |
|  | G17NL508 G17NL554 G17NL459 G17NL472 G17NL476 G17NL582 G16NL737 G16NL748 G16NL760 |
|  | G16NL808 G16NL817 G16NL915 G16NL838 G16NL847 G16NL878 G16NL883 G16NL896 G17NL627 |
|  | G17NL379 G17NL393 G17NL416 G17NL426 G17NL433 G17NL642 G17NL656 G17NL658 G17NL475 |
|  | G17NL486 G17NL501 G17NL506 G17NL518 G17NL543 G17NL546 G17NL558 G17NL590 G17NL600 |
|  | G17NL613 G16NL730 G16NL743 G16NL746 G16NL755 G16NL770 G16NL788 G16NL792 G16NL805 |
|  | G16NL852 G16NL874 G16NL876 G16NL890 G16NL894 G16NL906 G16NL909 G17NL376 G17NL383 |
|  | G17NL400 G17NL405 G17NL419 G17NL641 G17NL650 G17NL654 G17NL662 G17NL473 G17NL535 |
|  | G17NL539 G17NL553 G17NL570 G17NL576 G17NL599 G17NL604 G17NL615 G17NL619 G18NL667 |
|  | G18NL675 G17NL504 G17NL512 G17NL519 G17NL528 G17NL534 G17NL579 G17NL583 G17NL596 |
|  | G17NL608 G17NL616 G16NL689 G16NL728 G16NL753 G16NL762 G16NL769 G16NL803 G16NL816 |
|  | G16NL823 G16NL829 G16NL837 G16NL850 G16NL921 G17NL390 G17NL413 G17NL429 G17NL633 |
|  | G17NL643 G17NL465 G17NL478 G17NL480 G17NL487 G17NL490 G17NL493 G17NL516 G17NL517 |
|  | G17NL520 G17NL544 G17NL545 G17NL552 G17NL555 G17NL571 G17NL572 G17NL585 G17NL603 |
|  | G17NL606 G16NL682 G16NL692 G16NL717 G16NL733 G16NL736 G16NL749 G16NL772 G16NL775 |
|  | G16NL781 G16NL782 G16NL786 G16NL848 G16NL869 G16NL882 G16NL892 G17NL628 G17NL389 |
|  | G17NL394 G17NL403 G17NL406 G17NL407 G17NL421 G17NL660 G17NL447 G17NL461 G17NL481 |
|  | G17NL498 G17NL507 G17NL530 G17NL564 G17NL573 G17NL618 G16NL685 G16NL696 G16NL699 |
|  | G16NL712 G16NL732 G16NL752 G16NL763 G16NL767 G16NL771 G16NL794 G16NL795 G16NL798 |
|  | G16NL844 G16NL854 G16NL855 G16NL864 G16NL880 G16NL899 G16NL907 G17NL397 G17NL423 |
|  | G17NL424 G17NL430 G17NL432 G17NL440 G17NL638 G17NL494 G17NL531 G17NL566 G17NL589 |
|  | G17NL605 G17NL612 G17NL617 G16NL677 G16NL688 G16NL693 G16NL695 G16NL754 G16NL761 |
|  | G16NL783 G16NL810 G16NL818 G16NL831 G16NL832 G16NL835 G16NL873 G16NL877 G16NL905 |
|  | G16NL911 G16NL917 G17NL626 G17NL386 G17NL398 G17NL409 G17NL431 G17NL434 G17NL661 |
|  | G17NL441 G17NL444 G17NL449 G17NL452 G17NL454 G17NL463 G17NL484 G17NL491 G17NL536 |
|  | G17NL538 G17NL569 G17NL577 G17NL601 G17NL607 G17NL614 G18NL666 G16NL680 G16NL687 |
|  | G16NL705 G16NL724 G16NL718 G16NL726 G16NL731 G16NL740 G16NL747 G16NL764 G16NL809 |
|  | G16NL813 G16NL822 G16NL827 G16NL885 G16NL900 G16NL910 G16NL912 G16NL919 G17NL380 |
|  | G17NL382 G17NL411 G17NL634 G17NL647 G17NL652 G17NL659 G17NL469 G17NL492 G17NL521 |
|  | G17NL523 G17NL526 G17NL532 G17NL541 G17NL547 G17NL548 G17NL557 G17NL593 G17NL594 |
|  | G17NL598 G17NL621 G17NL622 G16NL679 G16NL683 G16NL703 G16NL711 G16NL720 G16NL779 |
|  | G16NL842 G16NL860 G16NL863 G16NL866 G16NL868 G16NL879 G16NL897 G16NL904 G17NL385 |
|  | G17NL427 G17NL629 G17NL640 G17NL455 G17NL474 G17NL482 G17NL483 G17NL524 G17NL542 |
|  | G17NL551 G17NL562 G17NL592 G17NL610 G17NL611 G16NL691 G16NL741 G16NL757 G16NL768 |
|  | G16NL774 G16NL777 G16NL804 G16NL830 G16NL833 G16NL841 G16NL867 G16NL875 G17NL387 |
|  | G17NL404 G17NL410 G17NL635 G17NL649 G17NL458 G17NL470 G17NL499 |
