# Supplementary material for: Genetic diversity and population structure analyses of tropical maize inbred lines using Single Nucleotide Polymorphism markers
Source: PLoS One. 2025 Jan 24;20(1):e0315463. doi: 10.1371/journal.pone.0315463 (PMC11760008; doi:10.1371/journal.pone.0315463)
Supplement: S1 File — (ZIP) [file pone.0315463.s001.zip › Supplementary Table 6.docx]

Supplementary Table 6. Contrasting maize lines selected with the nearest and furthest distances among the 182 founder inbred lines based on pairwise genetic comparisons using SNP markers.

| **Line 1** | **Line 2** | **Genetic distance (centimorgans (cM))** |
| --- | --- | --- |
| Selection of lines with nearest genetic distances |  |  |
| 16AG16786 | 16AG16785 | 0.006 |
| 16AG16802 | 16AG16801 | 0.013 |
| 16AG16791 | 16AG16793 | 0.014 |
| 16AG16795 | 16AG16794 | 0.014 |
| 16AG16809 | 16AG16810 | 0.014 |
| 16AG16790 | 16AG16791 | 0.014 |
| 16AG16810 | 16AG16809 | 0.014 |
| GS-PL17 | GS-PL53 | 0.021 |
| GS-PL40 | GS-PL39 | 0.026 |
| 16AG16785 | 16AG16786 | 0.04 |
| GL16828OS | 16AG16813 | 0.046 |
| 15NN166W | 15AG146 | 0.055 |
| 15NN117W | 15AG113 | 0.063 |
| 15AG149 | 15AG150 | 0.067 |
| 15AG109 | 15AG108 | 0.071 |
| 15AG108 | 15AG110 | 0.071 |
| 16AG16815 | GS-PL26 | 0.072 |
| 16AG16803 | 16AG16807 | 0.073 |
| 15AG171 | GS-PL08 | 0.075 |
| 16AG16783 | 16AG16784 | 0.077 |
| 15AG152 | 15AG153 | 0.078 |
| 15AG172 | 15AG173 | 0.078 |
| 16AG16811 | 16AG16812 | 0.079 |
| 16AG16789 | 16AG16790 | 0.085 |
| GS-PL05 | 15AG115 | 0.086 |
| 16AG16803 | 16AG16804 | 0.087 |
| 16AG16796 | 16AG16810 | 0.098 |
| 15AG127 | 15AG128 | 0.104 |
| 16AG16816 | 16AG16815 | 0.105 |
| 15AG117 | GS-PL04 | 0.111 |
| 15AG144 | 15AG152 | 0.111 |
| 16AG179 | GS-PL15 | 0.112 |
| 15AG129 | 15AG130 | 0.113 |
| 16AG16817 | GS-PL26 | 0.114 |
| 15AG162 | 15AG163 | 0.114 |
| GS-PL07 | 15AG160 | 0.123 |
| 16AG16797 | 16AG16799 | 0.123 |
| 15AG143 | 15AG152 | 0.124 |
| 15AG174 | 15AG175 | 0.127 |
| 15AG126 | 15AG132 | 0.128 |
| 15AG140 | 15AG146 | 0.131 |
| GS-PL43 | GS-PL08 | 0.134 |
| 16AG16808 | GS-PL49 | 0.134 |
| GS-PL65 | GS-PL49 | 0.134 |
| 16AG16800 | 16AG16799 | 0.136 |
| 15AG131 | 15AG125 | 0.138 |
| 15AG124 | 15AG126 | 0.142 |
| GS-PL62 | GS-PL09 | 0.147 |
| 15AG133 | 15AG127 | 0.147 |
| 16AG16798 | 16AG16803 | 0.149 |
| Selection of lines with the furthest genetic distances |  |  |
| 15AG112 | GS-PL44 | 0.425 |
| 15AG169 | GS-PL44 | 0.425 |
| GS-PL28 | GS-PL44 | 0.425 |
| 16AG16791 | GS-PL44 | 0.425 |
| GS-PL07 | GS-PL44 | 0.425 |
| GS-PL47 | GS-PL44 | 0.425 |
| GS-PL44 | 15AG125 | 0.425 |
| GS-PL44 | GS-PL06 | 0.425 |
| GS-PL44 | 15AG109 | 0.425 |
| GS-PL44 | 16AG16812 | 0.425 |
| GS-PL44 | GS-PL69 | 0.425 |
| GS-PL44 | 15AG153 | 0.425 |
| GS-PL44 | 15AG146 | 0.425 |
| GS-PL22 | GS-PL44 | 0.426 |
| 16AG16792 | GS-PL44 | 0.426 |
| 15AG144 | GS-PL44 | 0.426 |
| 15AG140 | GS-PL44 | 0.426 |
| GS-PL59 | GS-PL44 | 0.426 |
| 16AG178 | GS-PL44 | 0.426 |
| 15AG133 | GS-PL44 | 0.426 |
| 15AG124 | GS-PL44 | 0.426 |
| GS-PL44 | 15AG143 | 0.426 |
| GS-PL44 | 15AG114 | 0.426 |
| GS-PL44 | GS-PL50 | 0.426 |
| GS-PL14 | GS-PL44 | 0.427 |
| 15AG128 | GS-PL44 | 0.427 |
| 15AG150 | GS-PL44 | 0.427 |
| 16AG16789 | GS-PL44 | 0.427 |
| 15AG148 | GS-PL44 | 0.427 |
| GS-PL44 | 15AG132 | 0.427 |
| GS-PL44 | 15AG131 | 0.427 |
| GS-PL44 | GS-PL58 | 0.427 |
| GS-PL44 | GS-PL16 | 0.427 |
| GS-PL44 | 15AG108 | 0.427 |
| GS-PL44 | 16AG16816 | 0.427 |
| GS-PL44 | 15AG123 | 0.427 |
| GS-PL37 | GS-PL44 | 0.428 |
| 15AG177 | GS-PL44 | 0.428 |
| 16AG16788 | GS-PL44 | 0.428 |
| 15AG162 | GS-PL44 | 0.428 |
| 15AG155 | GS-PL44 | 0.428 |
| 16AG16790 | GS-PL44 | 0.428 |
| 15AG115 | GS-PL44 | 0.428 |
| GS-PL01 | GS-PL44 | 0.428 |
| GS-PL44 | GS-PL24 | 0.428 |
| GS-PL44 | 15AG174 | 0.428 |
| GS-PL71 | GS-PL44 | 0.429 |
| 15AG134 | GS-PL44 | 0.429 |
| 15AG126 | GS-PL44 | 0.43 |
| 16AG16811 | GS-PL44 | 0.431 |
| GS-PL54 | GS-PL44 | 0.431 |
| GS-PL44 | 15AG149 | 0.431 |
| 15AG152 | GS-PL44 | 0.432 |
| GS-PL33 | GS-PL44 | 0.435 |
