# Supplementary material for: Genetic diversity and population structure analyses of tropical maize inbred lines using Single Nucleotide Polymorphism markers
Source: PLoS One. 2025 Jan 24;20(1):e0315463. doi: 10.1371/journal.pone.0315463 (PMC11760008; doi:10.1371/journal.pone.0315463)
Supplement: S1 File — (ZIP) [file pone.0315463.s001.zip › Supplementary Table 7.docx]

Supplementary Table 7. Contrasting maize lines selected with the nearest and furthest distances among the 866 derived inbred lines based on pairwise genetic comparisons using SNP markers.

| **Line 1** | **Line 2** | **Genetic distance**  **(centimorgans (cM))** |
| --- | --- | --- |
| Selection of lines with nearest genetic distances |  |  |
| G17NL211 | G17NL210 | 0.004 |
| G17NL473 | G17NL472 | 0.005 |
| G17NL194 | G16NL910 | 0.008 |
| G16NL804 | G16NL806 | 0.008 |
| G16NL854 | G16NL853 | 0.009 |
| G16NL853 | G16NL855 | 0.009 |
| G17NL602 | G17NL603 | 0.011 |
| G16NL895 | G16NL896 | 0.011 |
| G16NL919 | G16NL920 | 0.015 |
| G16NL805 | G16NL806 | 0.015 |
| G16NL894 | G16NL895 | 0.016 |
| G18NL261 | G18NL260 | 0.017 |
| G17NL626 | G17NL625 | 0.019 |
| G17NL159 | G17NL557 | 0.019 |
| G17NL557 | G17NL159 | 0.019 |
| G17NL215 | G17NL216 | 0.02 |
| G17NL635 | G17NL636 | 0.021 |
| G16NL759 | G16NL758 | 0.021 |
| G16NL880 | G16NL879 | 0.021 |
| G17NL201 | G17NL200 | 0.022 |
| G16NL891 | G16NL893 | 0.023 |
| G16NL58 | G16NL792 | 0.023 |
| G16NL824 | G16NL825 | 0.023 |
| G16NL709 | G16NL708 | 0.024 |
| G16NL748 | G16NL747 | 0.024 |
| G16NL799 | G16NL800 | 0.025 |
| G16NL881 | G16NL879 | 0.025 |
| G16NL899 | G16NL900 | 0.025 |
| G17NL409 | G17NL408 | 0.026 |
| G17NL504 | G17NL505 | 0.026 |
| G17NL480 | G17NL481 | 0.027 |
| G16NL148 | G16NL149 | 0.027 |
| G16NL731 | G16NL730 | 0.029 |
| G16NL730 | G16NL732 | 0.029 |
| G16NL115 | G18NL279 | 0.03 |
| G18NL266 | G18NL267 | 0.03 |
| G16NL768 | G16NL769 | 0.03 |
| G16NL700 | G16NL701 | 0.03 |
| G16NL778 | G16NL779 | 0.03 |
| G16NL99 | G16NL100 | 0.032 |
| G17NL660 | G17NL661 | 0.032 |
| G16NL780 | G16NL781 | 0.034 |
| G16NL892 | G16NL893 | 0.034 |
| G16NL723 | G16NL724 | 0.036 |
| G16NL863 | G16NL864 | 0.036 |
| G16NL817 | G16NL818 | 0.036 |
| G16NL842 | G16NL843 | 0.037 |
| G16NL874 | G16NL875 | 0.037 |
| G16NL915 | G16NL834 | 0.037 |
| G17NL241 | G17NL242 | 0.038 |
| Selection of lines with the furthest genetic distances |  |  |
| G15NL342 | G15NL302 | 0.267 |
| G15NL326 | G15NL321 | 0.267 |
| G15NL298 | G15NL336 | 0.267 |
| G15NL344 | G15NL301 | 0.268 |
| G16NL54 | G15NL316 | 0.27 |
| G16NL62 | G16NL789 | 0.271 |
| G15NL285 | G16NL65 | 0.271 |
| G16NL691 | G15NL298 | 0.271 |
| G15NL294 | G16NL682 | 0.273 |
| G15NL288 | G15NL298 | 0.273 |
| G15NL293 | G15NL295 | 0.273 |
| G15NL360 | G15NL307 | 0.274 |
| G15NL08 | G15NL307 | 0.276 |
| G15NL348 | G15NL360 | 0.277 |
| G16NL912 | G16NL899 | 0.277 |
| G16NL886 | G15NL31 | 0.278 |
| G15NL312 | G15NL306 | 0.279 |
| G15NL325 | G15NL324 | 0.28 |
| G16NL144 | G16NL86 | 0.28 |
| G15NL332 | G15NL328 | 0.281 |
| G15NL333 | G15NL354 | 0.282 |
| G15NL317 | G15NL306 | 0.283 |
| G15NL300 | G17NL237 | 0.284 |
| G15NL323 | G15NL312 | 0.287 |
| G15NL289 | G15NL285 | 0.288 |
| G15NL286 | G18NL262 | 0.288 |
| G15NL365 | G15NL341 | 0.289 |
| G15NL287 | G15NL297 | 0.291 |
| G15NL318 | G15NL306 | 0.291 |
| G15NL310 | G15NL313 | 0.293 |
| G15NL319 | G15NL369 | 0.293 |
| G15NL334 | G15NL291 | 0.294 |
| G15NL355 | G15NL301 | 0.298 |
| G15NL292 | G15NL284 | 0.299 |
| G15NL327 | G15NL353 | 0.301 |
| G15NL303 | G15NL357 | 0.303 |
| G15NL349 | G15NL310 | 0.31 |
| G15NL337 | G15NL312 | 0.336 |
